# Supplementary material for: Determinants of Suicidality in the European General Population: A Systematic Review and Meta-Analysis
Source: Int J Environ Res Public Health. 2020 Jun 9;17(11):4115. doi: 10.3390/ijerph17114115 (PMC7312422; doi:10.3390/ijerph17114115)
Supplement: Supplementary file 1 [file ijerph-17-04115-s001.zip › Supplementary data/Tables/Table S1. Summary of variables used to characterize the articles included..docx]

**Table S1**. Summary of variables used to characterize the articles included.

| **Heading** | **Description** | **Categories** |
| --- | --- | --- |
| Author/s | Name of the first author, or the two authors, of each article |  |
| Year | Year of publication of each article | - 2008-2017 |
| Country | Name of the European country in which the study was conducted | - Belgium, Bulgaria, England, Finland, France, Germany, Great Britain, Greece, Northern Ireland, Italy, Latvia, The Netherlands, Portugal, Romania, Spain, Sweden, Turkey |
| Age range | Age range of the population sample studies | - 14-100 |
| Studies quality rating | Quality of the original study according to its assessment with an adapted version of the Quality Assessment Tool for Quantitative Studies [58] | - Strong - Moderate - Weak |
| Suicidality type | Classification of suicidal behaviors as outcome analyzed in meta-analysis. When several behaviors are included in a single category in any of the reviewed articles, we have classified it according to the most severe behavior | **Death wishes**   - Death wishes - Mild suicidal behavior, referring to death wishes plus life weariness/tiredness of life   **Suicidal ideation (ideation)**   - Suicidal ideation/ thought/ thinking - Suicidal ideation/ thought/ thinking plus death wishes and/ or tiredness of life)   **Suicidal plans (plans)**   - Suicidal plans - Suicidal ideation/ thought/ thinking plus suicidal plan   **Suicidal attempts (attempts)**   - Suicidal attempts - Suicidal ideation/ thought/ thinking plus suicidal attempt - Serious suicidal behavior, referring to ideation/ thought/ thinking plus suicidal plan plus suicidal attempt)   **All suicidality**   - All specific suicidality as a group. |
| Period of time for suicidality | The period of time used to estimate the suicidal behaviors analyzed in meta-analysis | - Point (previous week or 2 weeks, previous month) - Previous 12 months (previous year) - Lifetime - All time periods (global) |
| Factors analyzed | Factors included in meta-analysis | **Demographic factors**:   - Gender (man / woman) - Age up to 35 years / rest - Age between 35 and 65 years / rest - Age over 65 years / rest - Relationship status: stable relationship (married, cohabitating, partnership) / no stable relationship (single, no cohabitating, divorced, widower) - Residential setting: rural/ urban - Nationality: native/ no native - Education: university studies (degree, master's degree) / no university studies (illiterate, literate, primary education, no qualification, secondary education, A-level) - Employment situation: active (economically active, employed full-time and part-time) / inactive (unemployed, student, temporarily or permanently sick/disabled, retired, or looking after children at home)   **Psychosocial factors:**   - Social support: high/low - Adulthood adversity: having experienced (or not) at least one traumatic event in adulthood (history of trauma, traumatic events including natural and man-made disasters and accidents, combat, war, and refugee experiences, sexual and interpersonal violence, witnessing or perpetrating violence, and death or trauma to a loved one) - Childhood adversity: having experienced (or not) at least one traumatic event in childhood (physical abuse, sexual abuse, psychological abuse, emotional or physical neglect, parental death, parent divorce, other parental loss, family violence, physical illness, bullying and financial adversity before the age of 18)   **Clinical factors:**   - Family history of mental disorder: yes/ no - Any affective disorder: having or not major depression, bipolar I disorder, bipolar II disorder, dysthymia or persistent depressive disorder - Major depression - Anxiety/stress/somatoform disorders: having or not anxiety disorder not otherwise specified, generalized anxiety disorder, agoraphobia, panic disorder, social phobia, specific phobia, obsessive compulsive disorder, posttraumatic stress disorder, adjustment disorder, depersonalization and any anxiety disorder - Substance use: frequent alcohol consumption (at least two times per week, hazardous consumption, alcohol abuse, alcohol dependence) and use or dependence of drugs (tobacco, cannabis, amphetamines, cocaine, ecstasy, LSD, tranquillizers, crack, and heroin) - Frequent alcohol consumption (at least two times per week, hazardous consumption, alcohol abuse, alcohol dependence) - Tobacco use: daily smoking - Any mental disorder: all disorders included in any affective disorders, anxiety, stress and somatoform disorders and substance use, in addition to personality dysfunction, sleep problems, somatic symptoms, attention deficit disorder and intermittent explosive disorder and others psychological syndromes - Body mass index: <30Kg/m^2^ or ≥30Kg/m^2^ |
| Assessment tools for demographic factors | Evaluation tools used in each study for demographic factors included in meta-analysis | - Computer-assisted personal interview - Computer-assisted telephone interview - Demographic data sheet - Face-to-face interview - Face-to-face questionnaires - Paper-and-pencil interview - Questionnaire ad hoc - Structured interview using computer-assisted personal interviewing |
| Assessment tools for psychosocial factors | Evaluation tools used in each study for the psychosocial factors included in meta-analysis | - Childhood Trauma Questionnaire (CTQ) [94] - Composite International Diagnostic Interview (CIDI) [73] - Face-to-face interview - Medical Outcome Study (MOS) Social Support Survey [78] - Questionnaire ad hoc - Stressful Life Events (SLE) [85] - Use of cards with options and interview |
| Assessment tools for clinical factors | Evaluation tools used in each study for the clinical factors included in meta-analysis | - 7-items Generalized Anxiety Disorder (GAD-7) [69] - Alcohol Use Disorders Identification Test (AUDIT) [64] - Beck Anxiety Inventory (BAI) [95] - Beck Depression Inventory (BDI) [96] - Cambridge Depersonalization Scale (CDS) [88] - Clinical Interview Schedule – Revised (CIS-R) [61] - Composite International Diagnostic Interview (CIDI) [73] - Computer-assisted personal interview - Face-to-face interview - Mini Social Phobia Inventory (Mini-SPIN) [87] - Patient Health Questionnaire (PHQ-9) [70] - Posttraumatic Stress Disorder Checklist (PCL-4) [62] - Questionnaire ad hoc - Standardized Assessment of Personality Abbreviated Scale (SAPAS) [63] - Structured Clinical Interview for DSM-5 (SCID-I) [66] - Type-D scale (DS14) [89] |
