# Supplementary material for: Determinants of Suicidality in the European General Population: A Systematic Review and Meta-Analysis
Source: Int J Environ Res Public Health. 2020 Jun 9;17(11):4115. doi: 10.3390/ijerph17114115 (PMC7312422; doi:10.3390/ijerph17114115)
Supplement: Supplementary file 1 [file ijerph-17-04115-s001.zip › Supplementary data/Tables/Table S10. Publication bias..docx]

**Table S10.** Publication bias.

| **Suicidality** | **Period of time** | **Factor** | **Egger’s Test** | **Trim and Fill** | | |
| --- | --- | --- | --- | --- | --- | --- |
|  |  |  |  | **Missing effect sizes** | **Adjusted OR^1^** | ***p*-value** |
| All suicidality | Global | Gender (woman) | z = 2.88 ;  p = 0 | 22 | 1.31  (1.17–1.46) | 0 |
| All suicidality | Global | Education (university studies) | z = 0.51 ;  p = 0.61 | 1 | 0.53  (0.11–2.51) | 0.4231 |
| All suicidality | Global | Age up to 35 years | z = -3.24 ; p = 0 | 2 | 1.43  (0.58–3.55) | 0.4392 |
| All suicidality | Global | Age between 35 and 65 years | z = 1.7 ;  p = 0.09 | 0 | 0.89  (0.39–2.03) | 0.7869 |
| All suicidality | Global | Anxiety/stress /somatoform disorders | z = -2.57 ; p = 0.01 | 2 | 4.67  (3.06–7.12) | 0 |
| All suicidality | Global | Frequent alcohol consumption | z = -2.08 ; p = 0.04 | 0 | 1.52  (0.63–3.7) | 0.3527 |
| All suicidality | Global | Tobacco use | z = 2.08 ;  p = 0.04 | 1 | 2.58  (2.06–3.23) | 0 |
| All suicidality | Global | Body mass index | z = 5.62 ;  p = 0 | 0 | 2.58  (1.13–5.89) | 0.0249 |
| Ideation | Global | Frequent alcohol consumption | z = -2.08 ; p = 0.04 | 0 | 1.52  (0.63–3.70) | 0.3527 |

^1^ 95% confidence interval.
