# Supplementary material for: Determinants of Suicidality in the European General Population: A Systematic Review and Meta-Analysis
Source: Int J Environ Res Public Health. 2020 Jun 9;17(11):4115. doi: 10.3390/ijerph17114115 (PMC7312422; doi:10.3390/ijerph17114115)
Supplement: Supplementary file 1 [file ijerph-17-04115-s001.zip › Supplementary data/Tables/Table S2. Adaptations made to the Quality Assessment Tool for Quantitative Studies..docx]

**Table S2**. Adaptations made to the Quality Assessment Tool for Quantitative Studies.

| Section | Adaptation |
| --- | --- |
| A) Selection bias | The evaluation was performed as indicated by the original instrument. Q1 question the representativeness of the target population and Q2 for the percentage of selected individuals that agreed to participate^1^. It was rated as *strong* when the selected individuals are representative of the target population and there was a minimum of 80% participation^1^. It was rated as *moderate* when the selected individuals are somewhat likely to be representative and there is 60–79% participation or participation is not described^1^. *Weak* was rated when representativeness is low and participation is less than 60% or both selection and level of participation are not described^1^. In the case of Q2, when the studies used different samples, the percentage of participation was calculated using an arithmetic mean. |
| B) Study design | The original source indicates that any non-experimental design is scored as *moderate*^1^. Taking into account that the objective we propose involves observational studies, we value as *moderate* when the randomization method is not described, while *strong* when it is described. |
| C) Cofounders | It does not apply in our study since we do not perform intervention or exposure. Therefore, it is indicated as *does not apply*. |
| D) Blinding | It does not apply in our study since it is not an experimental study. Therefore, it is indicated as *does not apply*. |
| E) Data collection methods | It was performed as indicated by the original instrument: *strong* when data collection tools are both valid and reliable, *moderate* when they are valid but they are not reliable o reliability is not described and *weak* when they are not valid or validity and reliability are not described^1^. |
| F) Withdrawals and drop-outs | It only applies to studies with follow-up, so only one study was assessed as indicated by the original source (*strong* with a follow-up rate of 80% or greater, *moderate* with a 60–79%, *weak* when is less than 60% or when it is not described)^1^. The rest of the studies were indicated as *does not apply*. |
| Global rating | It has been classified as *weak*, *strong* or *moderate* when all sections had the same assessment. In other combinations, it was rated as *moderate*. |

^1^ Extracted from Quality Assessment Tool for Quantitative Studies [58].
