# Supplementary material for: Determinants of Suicidality in the European General Population: A Systematic Review and Meta-Analysis
Source: Int J Environ Res Public Health. 2020 Jun 9;17(11):4115. doi: 10.3390/ijerph17114115 (PMC7312422; doi:10.3390/ijerph17114115)
Supplement: Supplementary file 1 [file ijerph-17-04115-s001.zip › Supplementary data/Tables/Table S3 Quality assessment for studies included in this meta-analysis.docx]

**Table S3: Quality assessment for studies included in this meta-analysis^1^.**

| Author/s (Year) | Factor A | | | Factor B | Factor C and D | Factor E | | | Factor F | Global quality |
| --- | --- | --- | --- | --- | --- | --- | --- | --- | --- | --- |
|  | Q1 | Q2 | Global |  |  | Q1 | Q2 | Global |  |  |
| Aschan et al. (2013) | Very likely | Less than 60% | Weak | Strong | Does not apply | Cannot tell | Cannot tell | Weak | Does not apply | Moderate |
| Atay et al. (2012) | Very likely | Cannot tell | Moderate | Strong | Does not apply | Cannot tell | Cannot tell | Weak | Does not apply | Moderate |
| Bebbington et al. (2009) | Very likely | 60–79% | Moderate | Strong | Does not apply | Cannot tell | Cannot tell | Weak | Does not apply | Moderate |
| Blüml et al. (2013) | Very likely | Less than 60% | Weak | Strong | Does not apply | Yes | Yes | Strong | Does not apply | Moderate |
| Boyd et al. (2015) | Very likely | 60–79% | Moderate | Strong | Does not apply | Cannot tell | Cannot tell | Weak | Does not apply | Moderate |
| Bruffaerts et al. (2015) | Very likely | Less than 60% | Weak | Strong | Does not apply | Yes | Yes | Strong | Does not apply | Moderate |
| Economou et al. (2016) | Very likely | 80–100% | Strong | Strong | Does not apply | Cannot tell | Cannot tell | Weak | Does not apply | Moderate |
| Economou et al. (2013) | Very likely | 80–100% | Strong | Strong | Does not apply | Yes | Yes | Strong | Does not apply | Strong |
| Forkmann et al. (2012) | Very likely | Less than 60% | Weak | Strong | Does not apply | Yes | Yes | Strong | Does not apply | Moderate |
| Gisle & Van Oyen (2013) | Very likely | 60–79% | Moderate | Strong | Does not apply | Cannot tell | Cannot tell | Weak | Does not apply | Moderate |
| Hintikka et al. (2009) | Very likely | 60–79% | Moderate | Moderate | Does not apply | Yes | Yes | Strong | Moderate | Moderate |
| Hiswåls et al. (2015) | Very likely | Less than 60% | Weak | Moderate | Does not apply | Cannot tell | Cannot tell | Weak | Does not apply | Weak |
| Kovess-Masfety et al. (2011) | Very likely | 60–79% | Moderate | Strong | Does not apply | Cannot tell | Cannot tell | Weak | Does not apply | Moderate |
| Lara et al. (2015) | Very likely | 60–79% | Moderate | Strong | Does not apply | Yes | Yes | Strong | Does not apply | Moderate |
| McDonald et al. (2017) | Very likely | Less than 60% | Weak | Strong | Does not apply | Cannot tell | Cannot tell | Weak | Does not apply | Moderate |
| Meltzer et al. (2011) | Very likely | Less than 60% | Weak | Strong | Does not apply | Cannot tell | Cannot tell | Weak | Does not apply | Moderate |
| Michal et al. (2010) | Very likely | 60–79% | Moderate | Strong | Does not apply | Yes | Yes | Strong | Does not apply | Moderate |
| Miret et al. (2014) | Very likely | 60–79% | Moderate | Strong | Does not apply | Yes | Yes | Strong | Does not apply | Strong |
| O’Neill et al. (2014) | Very likely | 60–79% | Moderate | Strong | Does not apply | Yes | Yes | Strong | Does not apply | Moderate |
| Rancāns et al. (2016) | Very likely | Less than 60% | Weak | Strong | Does not apply | Cannot tell | Cannot tell | Weak | Does not apply | Moderate |
| Saraçli et al. (2016) | Very likely | 80–100% | Strong | Strong | Does not apply | Yes | Yes | Strong | Does not apply | Strong |
| Scocco et al. (2008) | Very likely | 60–79% | Moderate | Strong | Does not apply | Yes | Yes | Strong | Does not apply | Moderate |
| Spiers et al. (2014) | Very likely | 60–79% | Moderate | Strong | Does not apply | Cannot tell | Cannot tell | Weak | Does not apply | Moderate |
| Tempier & Guérin (2015) | Very likely | Less than 60% | Weak | Strong | Does not apply | Yes | Yes | Strong | Does not apply | Moderate |
| Ten Have et al. (2013) | Very likely | 60–79% | Moderate | Strong | Does not apply | Yes | Yes | Strong | Does not apply | Moderate |
| Wagner et al. (2013) | Very likely | Less than 60% | Weak | Strong | Does not apply | Yes | Yes | Strong | Does not apply | Moderate |

^1^ Study quality according to an adapted version of the Quality Assessment Tool for Quantitative Studies [58].
