# Supplementary material for: Determinants of Suicidality in the European General Population: A Systematic Review and Meta-Analysis
Source: Int J Environ Res Public Health. 2020 Jun 9;17(11):4115. doi: 10.3390/ijerph17114115 (PMC7312422; doi:10.3390/ijerph17114115)
Supplement: Supplementary file 1 [file ijerph-17-04115-s001.zip › Supplementary data/Tables/Table S4. Sensitivity analysis results in cases of reduction of more than 5% considering all time periods..docx]

**Table S4.** Sensitivity analysis results in cases of reduction of more than 5% considering all time periods.

| **Factor** | **Suicidal behavior** | **Author/s and year**^1^ | **OR**^2^ | | **Heterogeneity**^3^ | |
| --- | --- | --- | --- | --- | --- | --- |
|  |  |  | **Before** | **After** | **Before** | **After** |
| Gender (woman) | All suicidality | Bebbington et al. (2009) | 1.56 | 1.58 | 87.65% | 78.53% |
| Gender (woman) | Ideation | Bebbington et al. (2009) | 1.36 | 1.44 | 91.19% | 69.23% |
| Gender (woman) | Plans | Boyd et al. (2015)g^4^ | 1.56 | 1.68 | 87.65% | 68.87% |
| Gender (woman) | Attempts | Boyd et al. (2015)j^5^ | 1.78 | 1.85 | 87.65% | 70.66% |
| Age over 65 years | All suicidality | Economou et al. (2013)a^6^ | 0.45 | 0.42 | 80.85% | 71.07% |
| Residential setting (urban) | All suicidality | Economou et al. (2016) | 0.69 | 0.83 | 93.89% | 85.09% |
| Education (university studies) | All suicidality | Hiswåls et al. (2015) | 1.79 | 0.76 | 98.81% | 46.83% |
| Social support (low) | All suicidality | Gisle & Van Oyen (2013) | 2.56 | 3.03 | 75.53% | 44.32% |
| Adulthood adversity | All suicidality | Bruffaerts et al. (2015) | 3.65 | 4.90 | 86.82% | 45.02% |
| Childhood adversity | All suicidality | Bruffaerts et al. (2015) | 3.53 | 4.01 | 91.12% | 83.85% |
| Any affective disorder | All suicidality | Michal et al. (2010) | 7.41 | 6.19 | 84.71% | 74.17% |
| Major depression | All suicidality | Michal et al. (2010) | 7.69 | 6.34 | 90.63% | 84.79% |
| Substance use | All suicidality | Michal et al. (2010) ALC^7^ | 2.45 | 2.57 | 84.84% | 76.15% |
| Substance use | Ideation | Michal et al. (2010) TOB^8^ | 2.18 | 2.34 | 82.94% | 63.29% |
| Substance use | Attempts | Tempier & Guérin (2015) THND^9^ | 3.26 | 3.65 | 73.58% | 65.64% |
| Tobacco use | All suicidality | Michal et al. (2010) | 2.67 | 2.83 | 84.87% | 75.58% |
| Any mental disorder | Death wishes | Atay et al. (2012) MD^10^ | 3.61 | 2.56 | 94.9% | 57.18% |

^1^ Study eliminated with sensitivity analysis. ^2^ Confidence interval of 95%. ^3^ Heterogeneity measured with I^2^. ^4^ Data from Portugal. ^5^ Data from The Netherlands. ^6^ 2009 Data. ^7^ Data on frequent alcohol consumption. ^8^ Data on use of tobacco. ^9^ Data on high nicotine dependence. ^10^ Data on major depression.
