# Supplementary material for: Determinants of Suicidality in the European General Population: A Systematic Review and Meta-Analysis
Source: Int J Environ Res Public Health. 2020 Jun 9;17(11):4115. doi: 10.3390/ijerph17114115 (PMC7312422; doi:10.3390/ijerph17114115)
Supplement: Supplementary file 1 [file ijerph-17-04115-s001.zip › Supplementary data/Tables/Table S5. Moderator analysis of period of time for all types of suicidality in demographic and psychosocial factors..docx]

**Table S5**. Moderator analysis of period of time for all types of suicidality in demographic and psychosocial factors.

| **Factor and period of time** | **OR (95% CI)^1^** | ***p*-value** | **Explained heterogeneity^2^** |
| --- | --- | --- | --- |
| Gender (woman) |  |  | 26.89% |
| Point^3^ | 0.97 (0.77–1.23) | 0.82 |  |
| 12-months | 1.37 (0.99–1.91) | 0.06 |  |
| Lifetime | 1.81 (1.39–2.34) | <0.05 |  |
| Age over 65 years |  |  | 0% |
| Point | 0.97 (0.50–1.86) | 0.92 |  |
| 12-months^3^ | 1.96 (1.18–3.26) | <0.05 |  |
| Lifetime | 1.47 (0.75–2.87) | 0.26 |  |
| Relationship status (stable) |  |  | 10.46% |
| Point | 1.80 (0.69–4.69) | 0.23 |  |
| 12-months^3^ | 0.86 (0.38–1.91) | 0.71 |  |
| Lifetime | 2.48 (0.92–6.71) | 0.07 |  |
| Residential setting (urban) |  |  | 11.97% |
| Point | 1.05 (0.33–3.33) | 0.93 |  |
| 12-months^3^ | 1.93 (0.80–4.68) | 0.14 |  |
| Lifetime | 0.48 (0.16–1.46) | 0.20 |  |
| Education (university studies) |  |  | 98.66% |
| 12-months^3^ | 0.05 (0.03–20.09) | <0.05 |  |
| Lifetime | 34.72 (18.28–65.96) | <0.05 |  |
| Employment situation (active) |  |  | 17.84% |
| Point | 0.32 (0.09–1.07) | 0.06 |  |
| 12-months^3^ | 4.22 (1.44–12.38) | <0.05 |  |
| Lifetime | 0.66 (0.19–2.24) | 0.50 |  |
| Social support (low) |  |  | 89.69% |
| Point | 1.07 (0.63–1.79) | 0.81 |  |
| 12-months^3^ | 0.28 (0.20–0.40) | <0.05 |  |
| Lifetime | 1.87 (1.23–2.83) | <0.05 |  |
| Childhood adversity |  |  | 0% |
| 12-months^3^ | 3.28 (0.60–17.94) | 0.17 |  |
| Lifetime | 1.08 (0.19–6.17) | 0.93 |  |

^1^ Weighted mean odds ratio with 95% confidence interval. ^2^ Heterogeneity explained with R^2^. ^3^ Moderator level used as a reference in the analysis.
