# Supplementary material for: Determinants of Suicidality in the European General Population: A Systematic Review and Meta-Analysis
Source: Int J Environ Res Public Health. 2020 Jun 9;17(11):4115. doi: 10.3390/ijerph17114115 (PMC7312422; doi:10.3390/ijerph17114115)
Supplement: Supplementary file 1 [file ijerph-17-04115-s001.zip › Supplementary data/Tables/Table S6. Moderator analysis of period of time for all types of suicidality in clinical factors..docx]

**Table S6**. Moderator analysis of period of time for all types of suicidality in clinical factors.

| **Factor and period of time** | **OR (95% CI)^1^** | ***p*-value** | **Explained heterogeneity^2^** |
| --- | --- | --- | --- |
| Any affective disorder |  |  | 90.44% |
| Point | 3.91 (2.29–6.69) | <0.05 |  |
| Lifetime^3^ | 4.58 (3.22–6.51) | <0.05 |  |
| Major depression |  |  | 90.84% |
| Point | 3.96 (2.28–6.86) | <0.05 |  |
| Lifetime^3^ | 4.53 (3.15–6.52) | <0.05 |  |
| Anxiety/stress/somatoform disorders |  |  | 72.73% |
| Point | 0.36 (0.13–0.98) | 0.04 |  |
| 12-months^3^ | 20.81 (8.23–52.62) | <0.05 |  |
| Lifetime | 0.13 (0.05–0.36) | <0.05 |  |
| Substance use |  |  | 13.89% |
| Point | 0.55 (0.33–0.92) | <0.05 |  |
| 12-months^3^ | 3.09 (2.20–4.34) | <0.05 |  |
| Lifetime | 0.83 (0.53–1.28) | 0.40 |  |
| Tobacco use |  |  | 9.28% |
| Point | 0.56 (0.31–1.03) | 0.06 |  |
| 12-months^3^ | 3.44 (2.36–5.03) | <0.05 |  |
| Lifetime | 0.74 (0.45–1.22) | 0.24 |  |
| Any mental disorder |  |  | 5.94% |
| Point | 1.46 (0.74–2.87) | 0.28 |  |
| 12-months^3^ | 3.63 (2.12–6.21) | <0.05 |  |
| Lifetime | 0.84 (0.46–1.53) | 0.57 |  |

^1^ Weighted mean odds ratio with 95% confidence interval. ^2^ Heterogeneity explained with R^2^. ^3^ Moderator level used as a reference in the analysis.
