# Supplementary material for: Determinants of Suicidality in the European General Population: A Systematic Review and Meta-Analysis
Source: Int J Environ Res Public Health. 2020 Jun 9;17(11):4115. doi: 10.3390/ijerph17114115 (PMC7312422; doi:10.3390/ijerph17114115)
Supplement: Supplementary file 1 [file ijerph-17-04115-s001.zip › Supplementary data/Tables/Table S7. Moderator analysis of period of time for suicidal ideation..docx]

**Table S7.** Moderator analysis of period of time for suicidal ideation.

| **Factor and period of time** | **OR (95% CI)^1^** | **p-value** | **Explained heterogeneity^2^** |
| --- | --- | --- | --- |
| Gender (woman) |  |  | 31.8% |
| Point^3^ | 0.92 (0.72–1.17) | 0.49 |  |
| 12-months | 1.43 (1.00–2.05) | 0.05 |  |
| Lifetime | 1.77 (1.33–2.36) | <0.05 |  |
| Anxiety/stress/somatoform disorders |  |  | 76.62% |
| Point | 0.35 (0.14–0.87) | <0.05 |  |
| 12-months^3^ | 20.81 (9.04–47.90) | <0.05 |  |
| Lifetime | 0.14 (0.05–0.38) | <0.05 |  |
| Substance use |  |  | 0% |
| Point | 0.66 (0.38–1.14) | 0.14 |  |
| 12-months^3^ | 2.57 (1.73–3.82) | <0.05 |  |
| Lifetime | 0.92 (0.54–1.59) | 0.78 |  |
| Any mental disorder |  |  | 0% |
| Point | 1.19 (0.54–2.65) | 0.66 |  |
| 12-months^3^ | 4.02 (2.06–7.83) | <0.05 |  |
| Lifetime | 0.79 (0.36–1.73) | 0.55 |  |

^1^ Weighted mean odds ratio with 95% confidence interval. ^2^ Heterogeneity explained with R^2^. ^3^ Moderator level used as a reference in the analysis.
