# Supplementary material for: Determinants of Suicidality in the European General Population: A Systematic Review and Meta-Analysis
Source: Int J Environ Res Public Health. 2020 Jun 9;17(11):4115. doi: 10.3390/ijerph17114115 (PMC7312422; doi:10.3390/ijerph17114115)
Supplement: Supplementary file 1 [file ijerph-17-04115-s001.zip › Supplementary data/Tables/Table S8. Moderator analysis of period of time for suicidal attempts..docx]

**Table S8**. Moderator analysis of period of time for suicidal attempts.

| **Factor and period of time** | **OR (95% CI)^1^** | ***p*-value** | **Explained heterogeneity^2^** |
| --- | --- | --- | --- |
| Gender (woman) |  |  | 0% |
| Point^3^ | 1.36 (0.69–2.68) | 0.37 |  |
| 12-months | 1.09 (0.44–2.67) | 0.86 |  |
| Lifetime | 1.39 (0.68–2.83) | 0.37 |  |
| Substance use |  |  | 30.4% |
| 12-months^3^ | 5.11 (2.77–9.44) | <0.05 |  |
| Lifetime | 0.55 (0.27–1.11) | 0.09 |  |
| Any mental disorder |  |  | 16.38% |
| Point | 6.15 (0.95–39.80) | 0.06 |  |
| 12-months | 2.99 (0.34–2.78) | <0.05 |  |
| Lifetime^3^ | 0.98 (0.34–2.78) | 0.96 |  |

^1^ Weighted mean odds ratio with 95% confidence interval. ^2^ Heterogeneity explained with R^2^. ^3^ Moderator level used as a reference in the analysis.
