# Supplementary material for: Determinants of Suicidality in the European General Population: A Systematic Review and Meta-Analysis
Source: Int J Environ Res Public Health. 2020 Jun 9;17(11):4115. doi: 10.3390/ijerph17114115 (PMC7312422; doi:10.3390/ijerph17114115)
Supplement: Supplementary file 1 [file ijerph-17-04115-s001.zip › Supplementary data/Tables/Table S9. Moderator analysis using period of time after sensibility analysis in gender considering woman as reference..docx]

**Table S9.** Moderator analysis using period of time after sensibility analysis in gender considering woman as reference.

| **Suicidality** | **Period of time** | **OR (95% CI)^1^** | ***p*-value** | **Explained heterogeneity^2^** |
| --- | --- | --- | --- | --- |
| All suicidality |  |  |  | 25.48% |
|  | Point^3^ | 1.16 (0.95–1.41) | 0.15 |  |
|  | 12-months | 1.15 (0.88–1.51) | 0.29 |  |
|  | Lifetime | 1.49 (1.20–1.85) | <0.05 |  |
| Suicidal ideation |  |  |  | 33.35% |
|  | Point^3^ | 1.15 (0.98–1.34) | 0.08 |  |
|  | 12-months | 1.16 (0.93–1.43) | 0.19 |  |
|  | Lifetime | 1.38 (1.16–1.65) | <0.05 |  |
| Suicidal plans |  |  |  | 0% |
|  | 12-months^3^ | 1.17 (0.50–2.47) | 0.71 |  |
|  | Lifetime | 1.48 (0.61–3.57) | 0.38 |  |
| Suicidal attempts |  |  |  | 0% |
|  | Point^3^ | 1.33 (0.74–2.38) | 0.34 |  |
|  | 12-months | 1.12 (0.52–2.44) | 0.77 |  |
|  | Lifetime | 1.49 (0.81–2.75) | 0.20 |  |

^1^ Weighted mean odds ratio with 95% confidence interval. ^2^ Heterogeneity explained with R^2^. ^3^ Moderator level used as a reference in the analysis.
