# Supplementary figures and images for: Determinants of Suicidality in the European General Population: A Systematic Review and Meta-Analysis
Source: Int J Environ Res Public Health. 2020 Jun 9;17(11):4115. doi: 10.3390/ijerph17114115 (PMC7312422; doi:10.3390/ijerph17114115)

# Desempleo

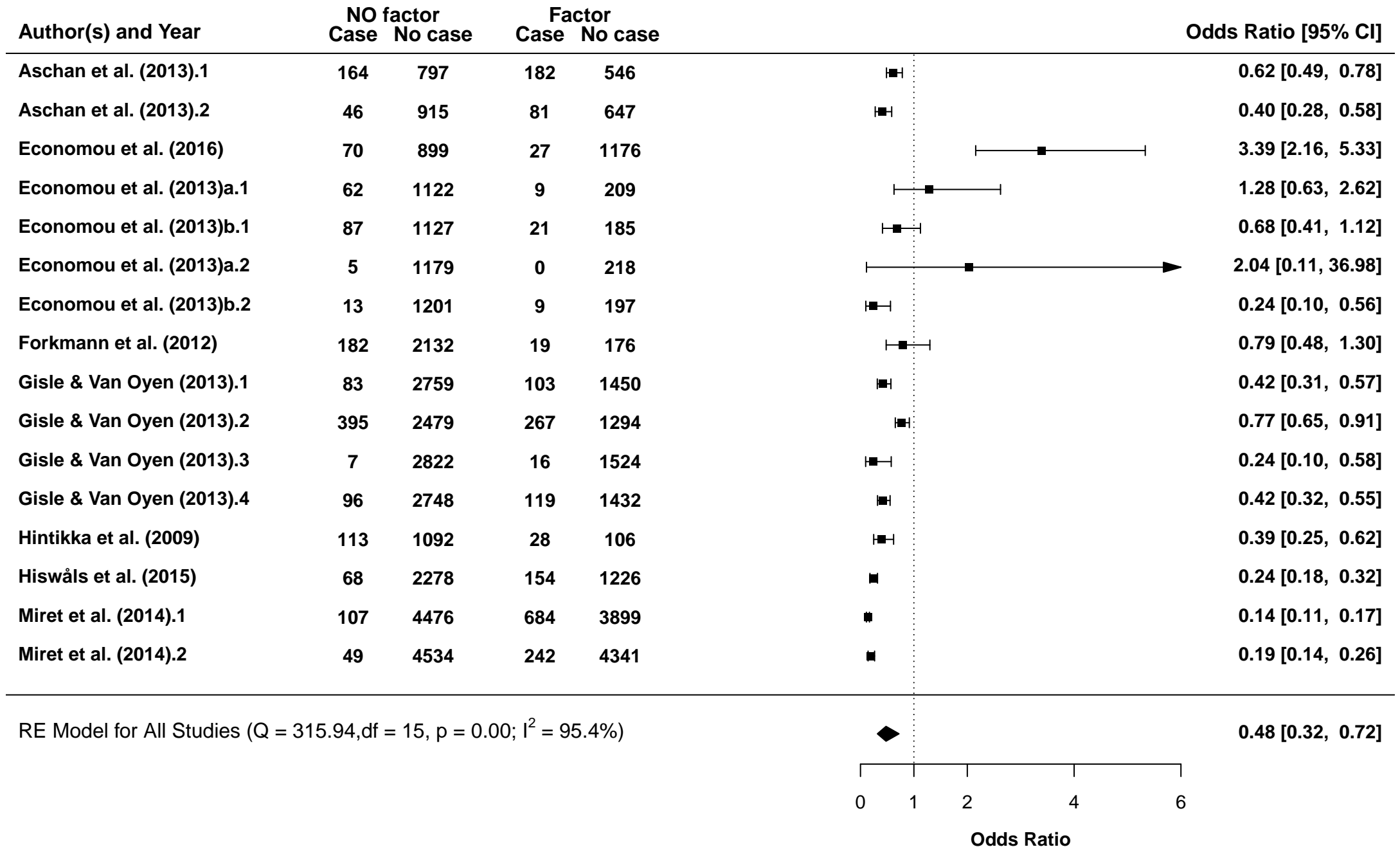

Supplement: Supplementary file 1 [file ijerph-17-04115-s001.zip › Supplementary data/Figures/Figure S10. Employment situation for all suicidality forest plot.pdf]

Apoyo social

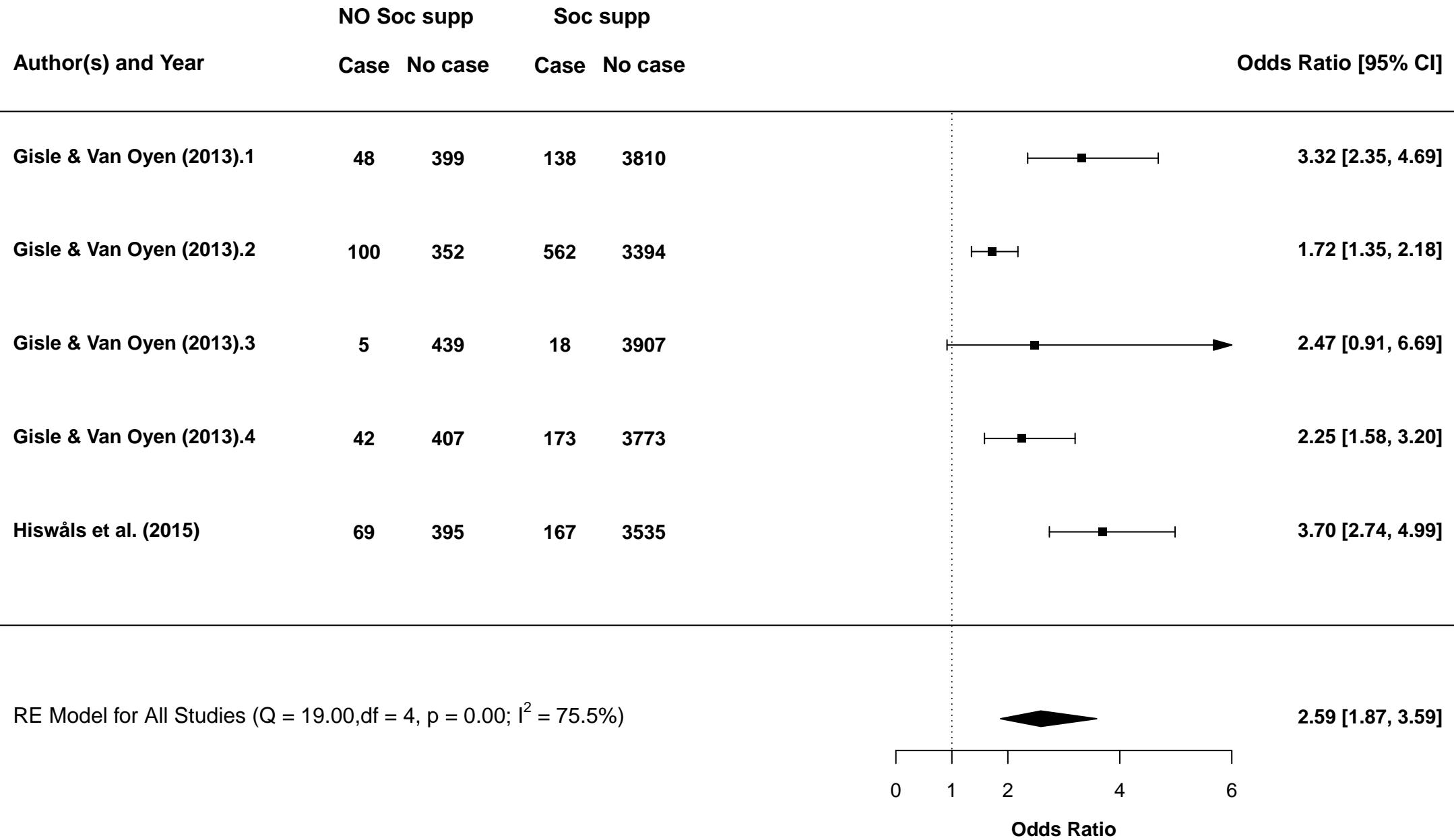

Supplement: Supplementary file 1 [file ijerph-17-04115-s001.zip › Supplementary data/Figures/Figure S11. Social support for all suicidality forest plot.pdf]

# traumas

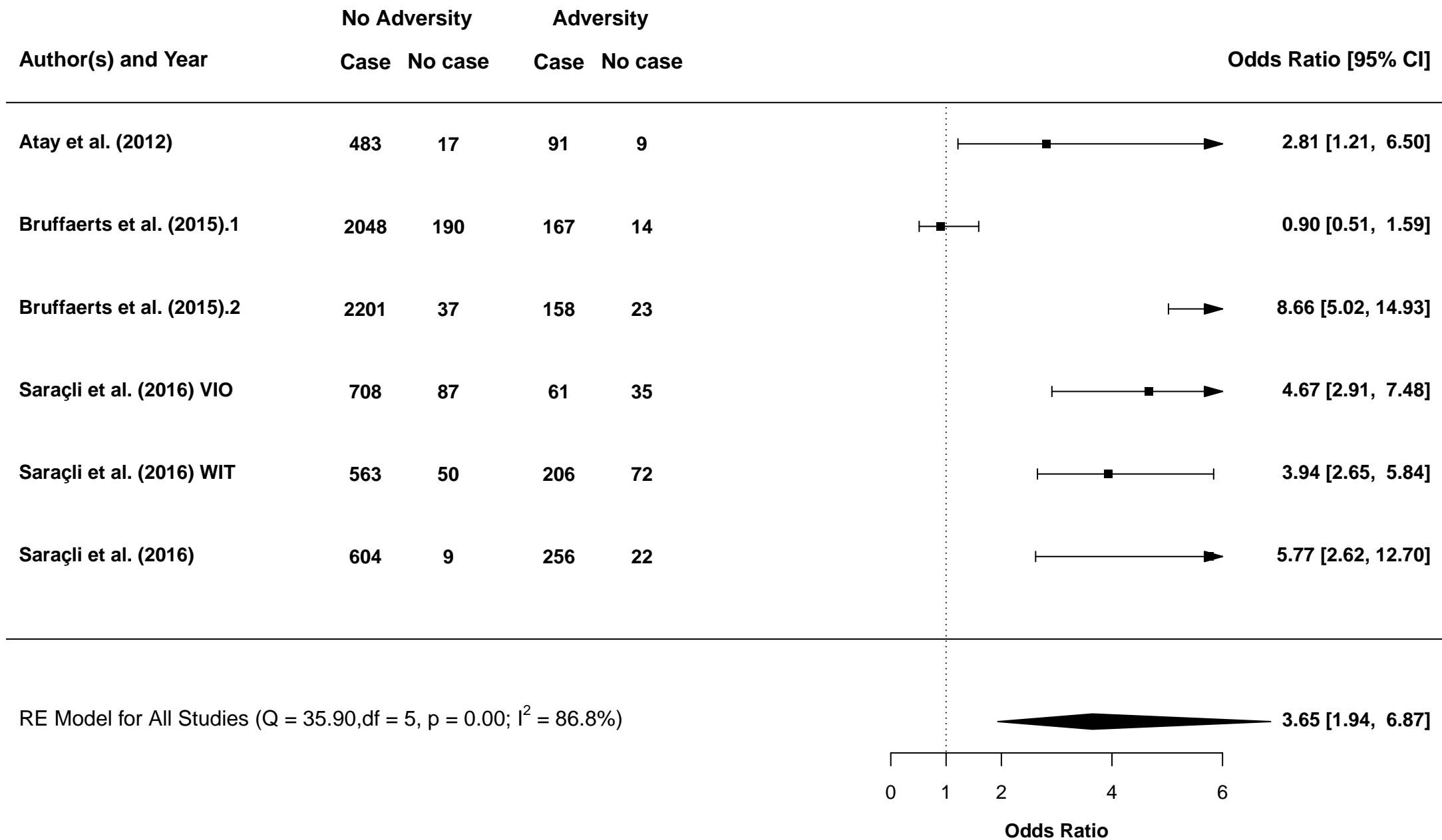

Supplement: Supplementary file 1 [file ijerph-17-04115-s001.zip › Supplementary data/Figures/Figure S12. Adulthood adversity for all suicidality forest plot.pdf]

# infancia

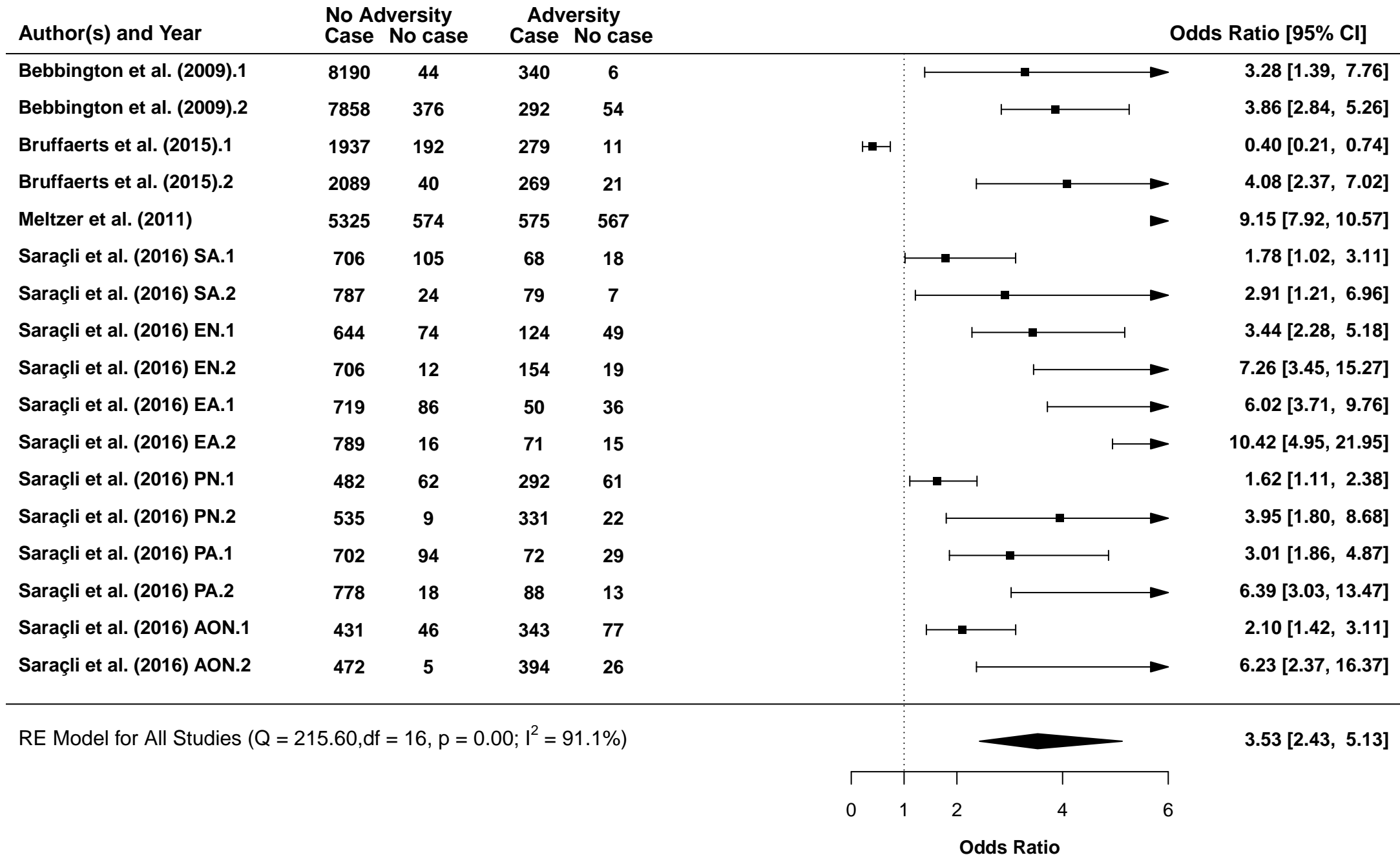

Supplement: Supplementary file 1 [file ijerph-17-04115-s001.zip › Supplementary data/Figures/Figure S13. Childhood adversity for all suicidality forest plot.pdf]

Todos los comportamientos suicidas.

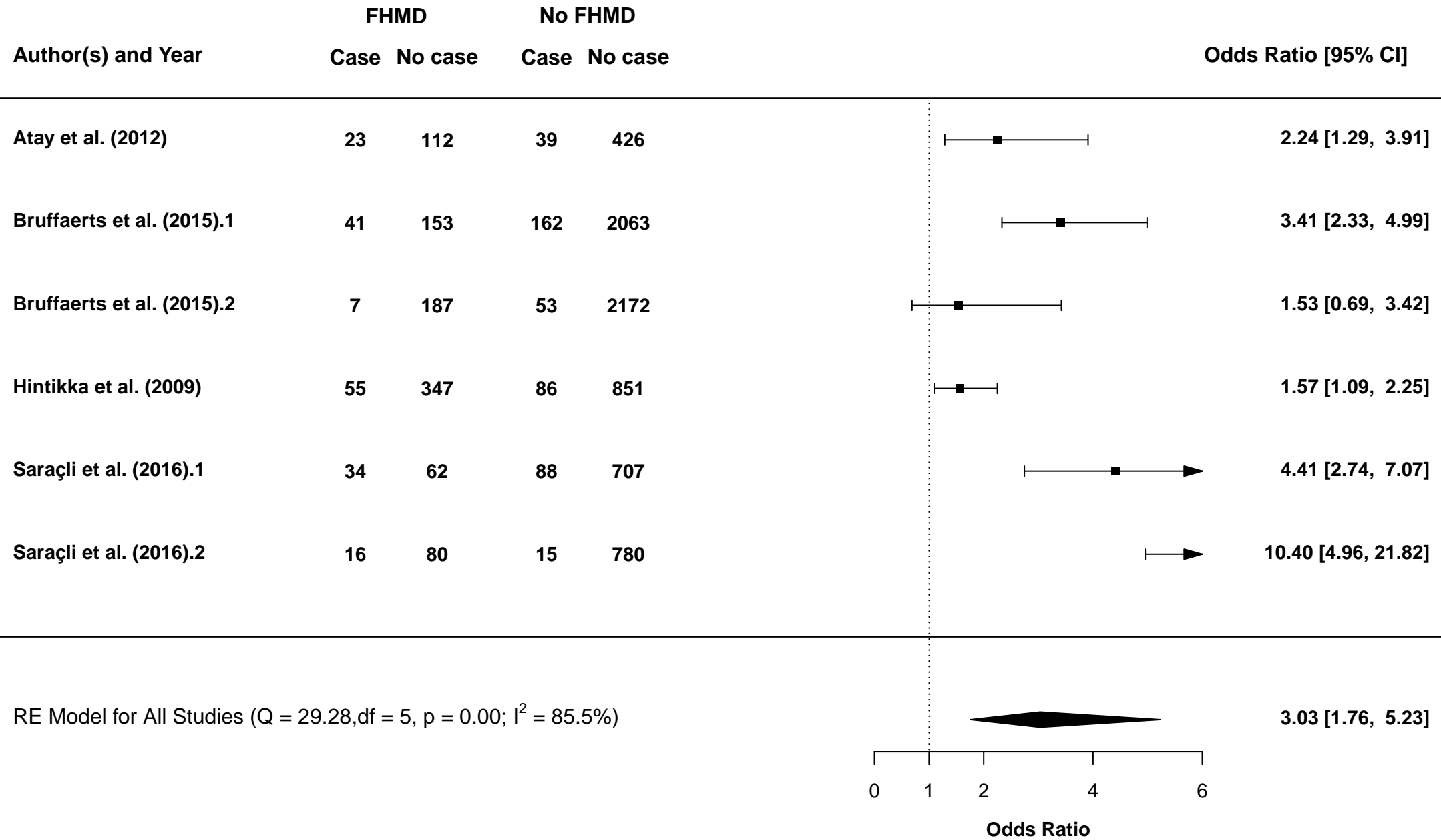

Supplement: Supplementary file 1 [file ijerph-17-04115-s001.zip › Supplementary data/Figures/Figure S14. Family history of mental disorder for all suicidality forest plot.pdf]

# Affective disorders

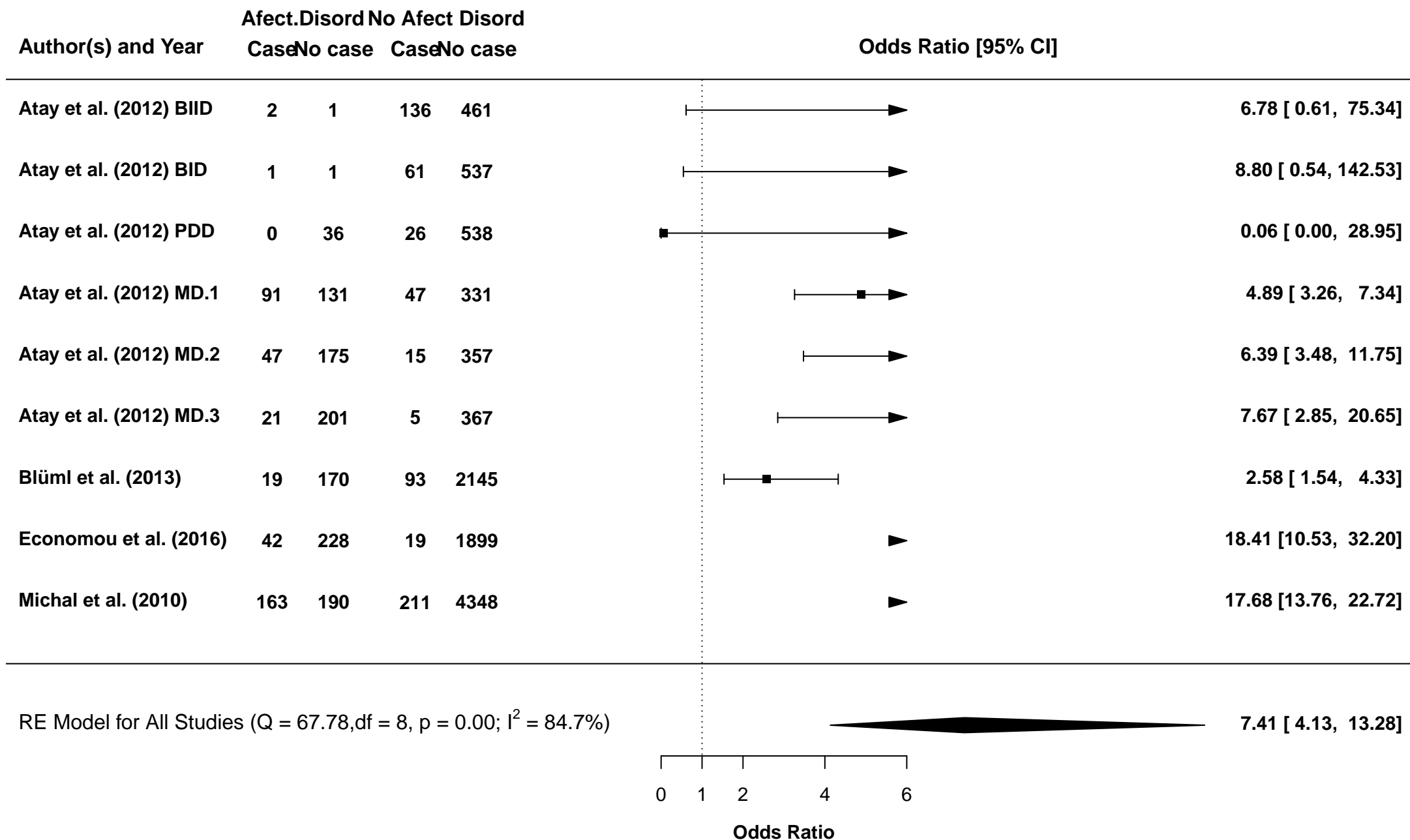

Supplement: Supplementary file 1 [file ijerph-17-04115-s001.zip › Supplementary data/Figures/Figure S15. Any affective disorder for all suicidality forest plot.pdf]

Depression

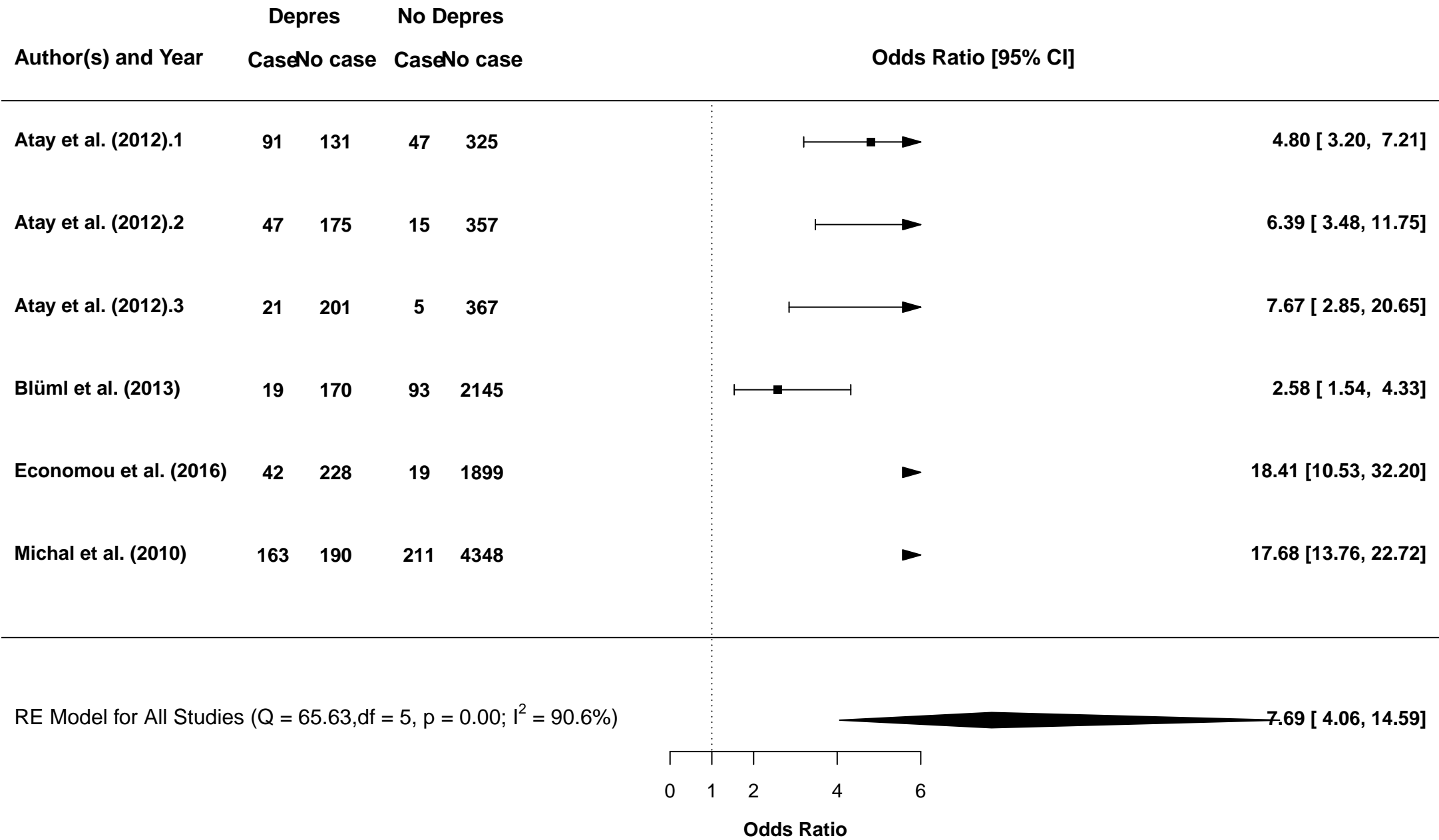

Supplement: Supplementary file 1 [file ijerph-17-04115-s001.zip › Supplementary data/Figures/Figure S16. Major depression for all suicidality forest plot.pdf]

# neuroticos

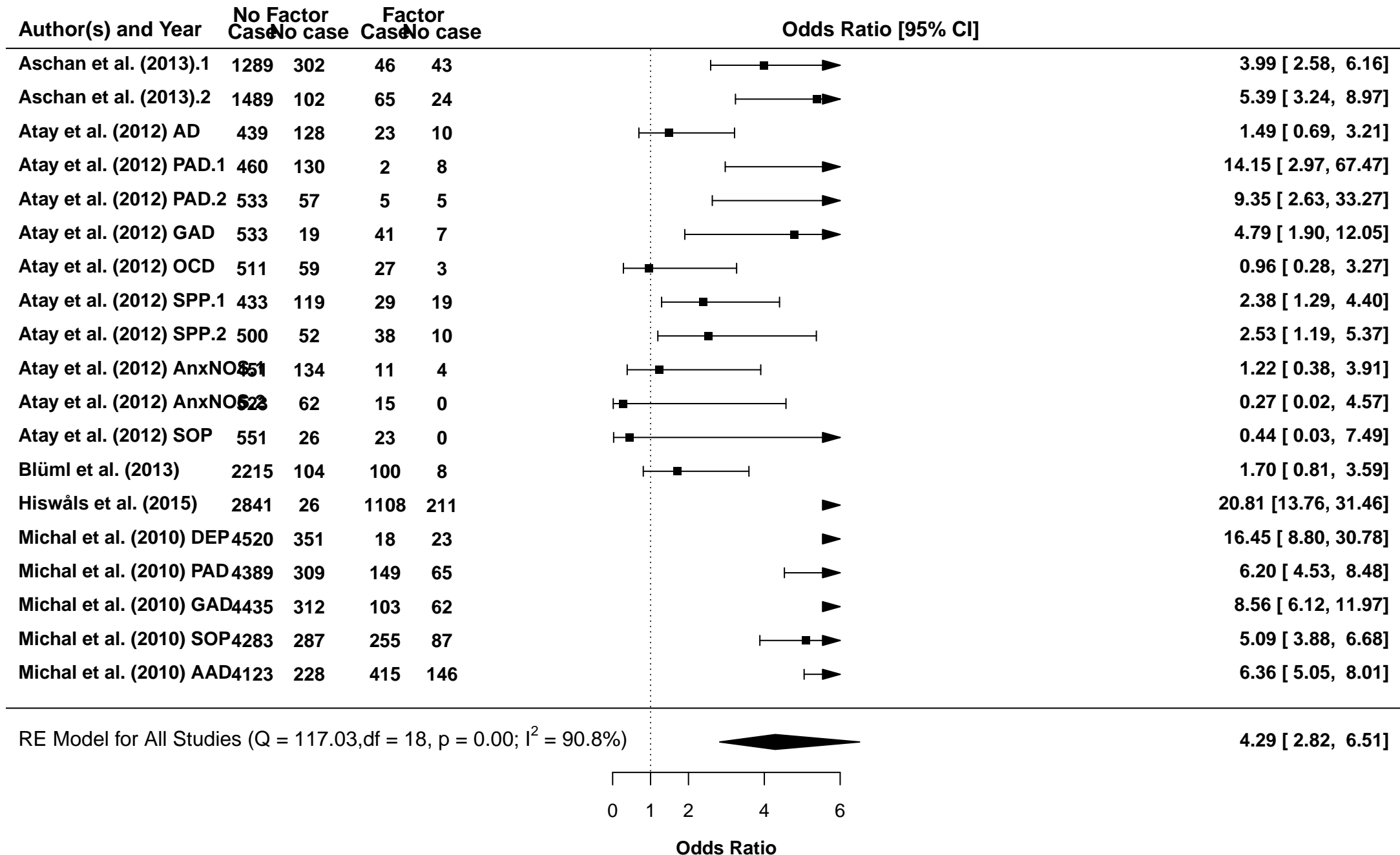

Supplement: Supplementary file 1 [file ijerph-17-04115-s001.zip › Supplementary data/Figures/Figure S17. Anxiety-stress-somatoform disorders for all suicidality forest plot.pdf]

## C. Sustancias

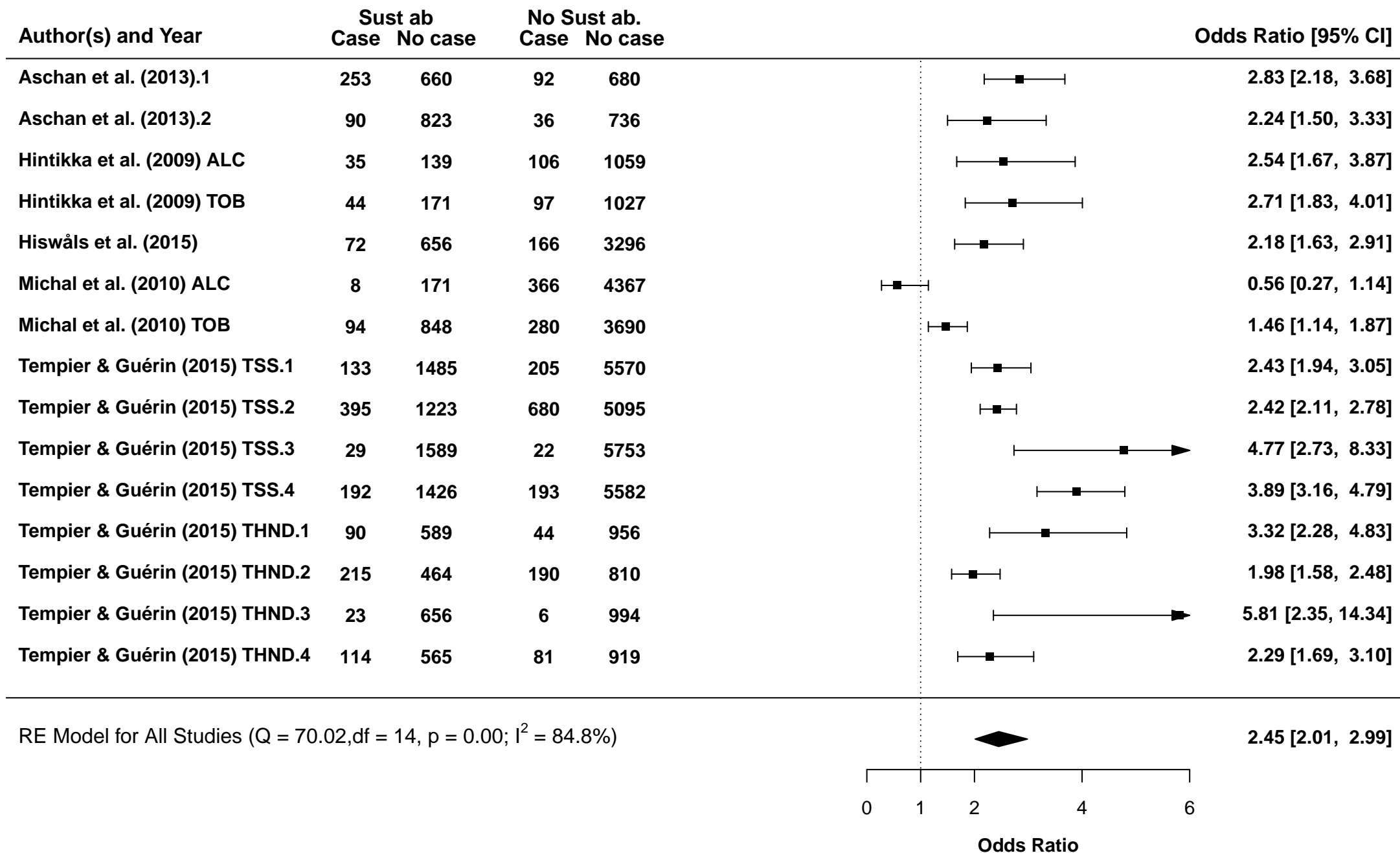

Supplement: Supplementary file 1 [file ijerph-17-04115-s001.zip › Supplementary data/Figures/Figure S18. Substance use for all suicidality forest plot.pdf]

alcohol

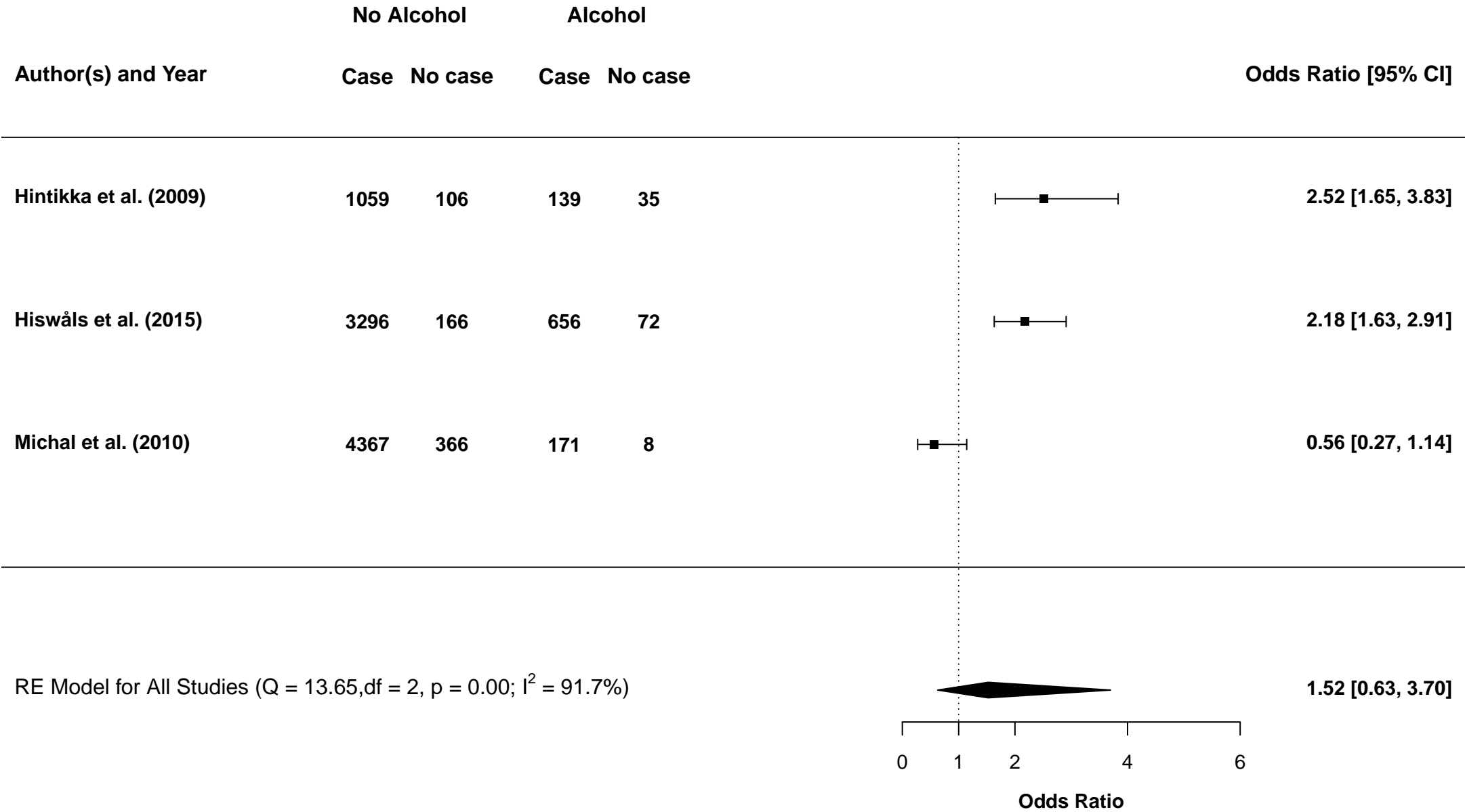

Supplement: Supplementary file 1 [file ijerph-17-04115-s001.zip › Supplementary data/Figures/Figure S19. Frequent alcohol consumption for all suicidality forest plot.pdf]

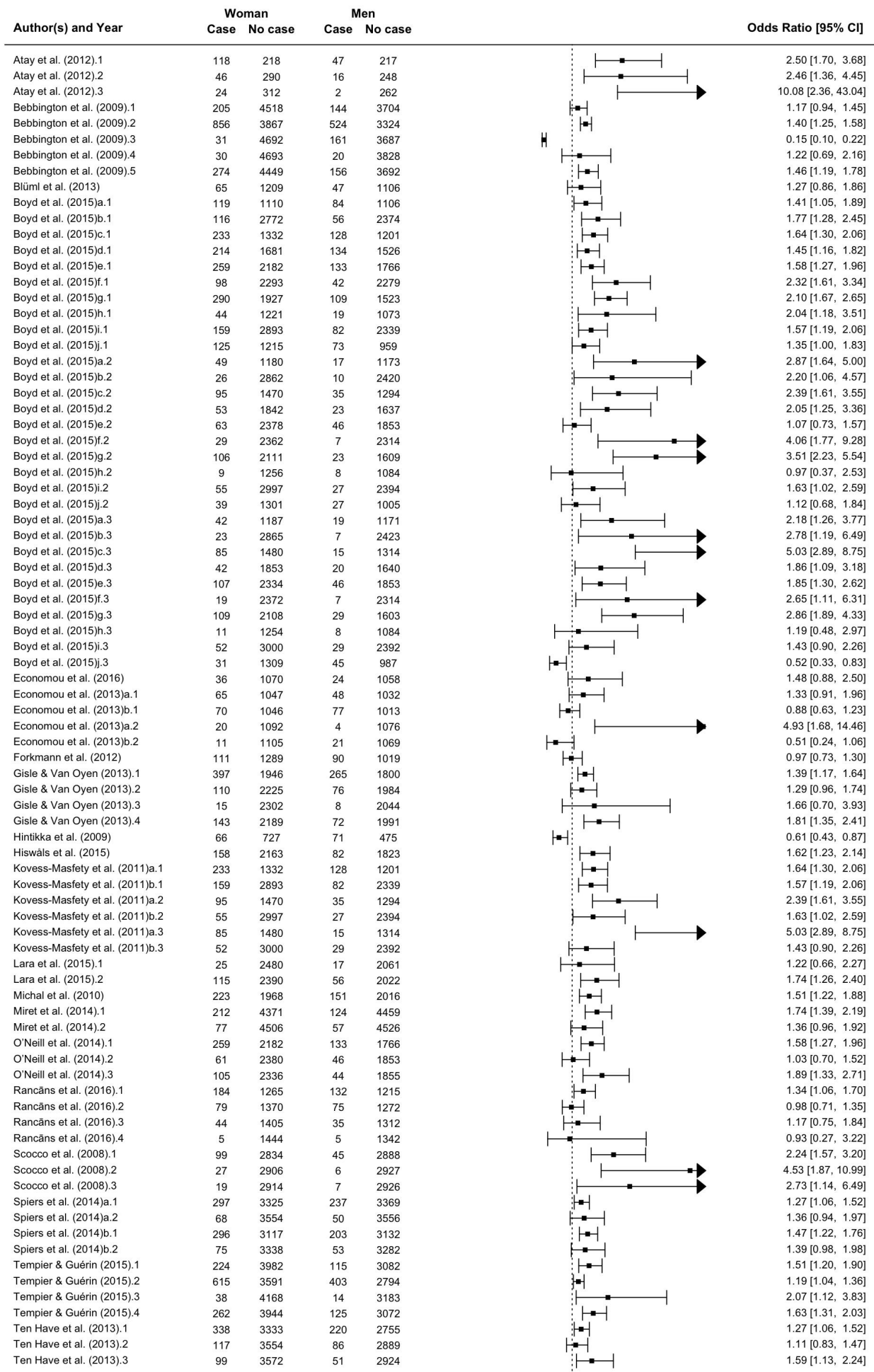

RE Model for All Studies (Q = 406.17,df = 82, p = 0.00; I<sup>2</sup> = 87.6%)

1.56 [1.40, 1.72]

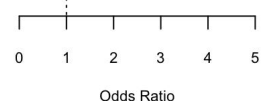

Supplement: Supplementary file 1 [file ijerph-17-04115-s001.zip › Supplementary data/Figures/Figure S2. Gender for all suicidality forest plot.pdf]

Tabaco

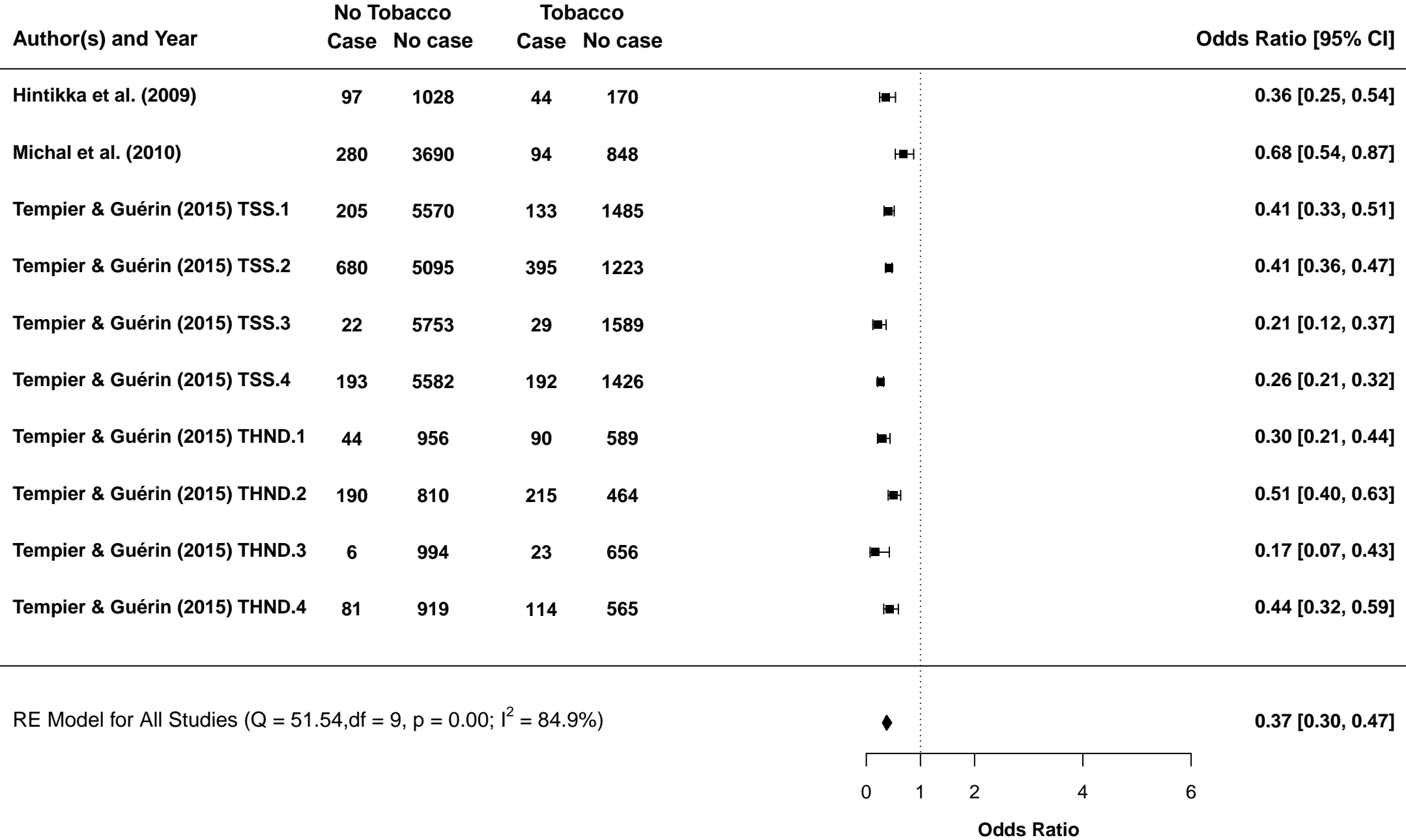

Supplement: Supplementary file 1 [file ijerph-17-04115-s001.zip › Supplementary data/Figures/Figure S20. Tobacco use for all suicidality forest plot.pdf]

# Tmental

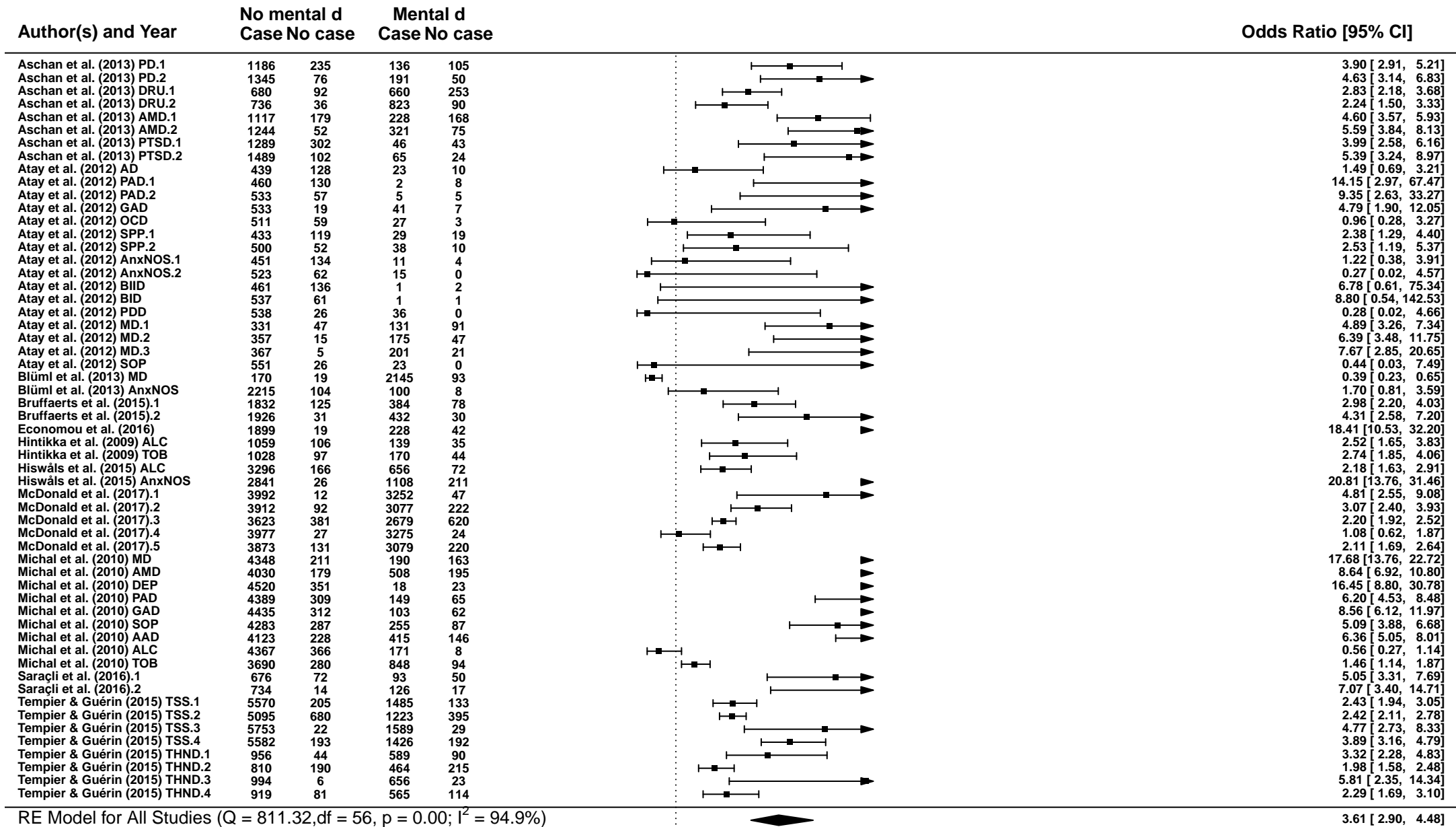

3.61 [ 2.90, 4.48]

Supplement: Supplementary file 1 [file ijerph-17-04115-s001.zip › Supplementary data/Figures/Figure S21. Mental disorders for all suicidality forest plot.pdf]

Obesidad

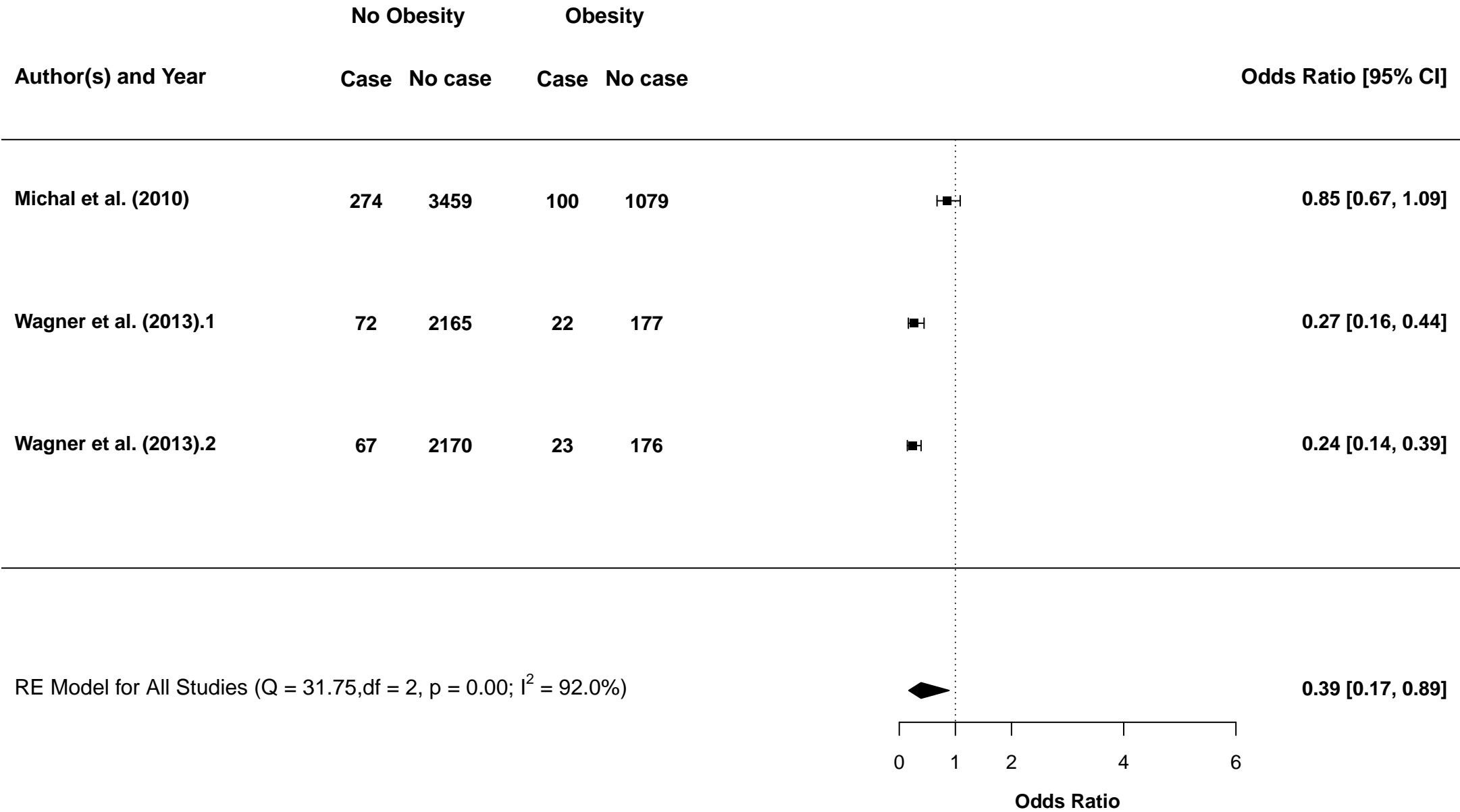

Supplement: Supplementary file 1 [file ijerph-17-04115-s001.zip › Supplementary data/Figures/Figure S22. Body mass index for all suicidality forest plot.pdf]

Edad menor de 35

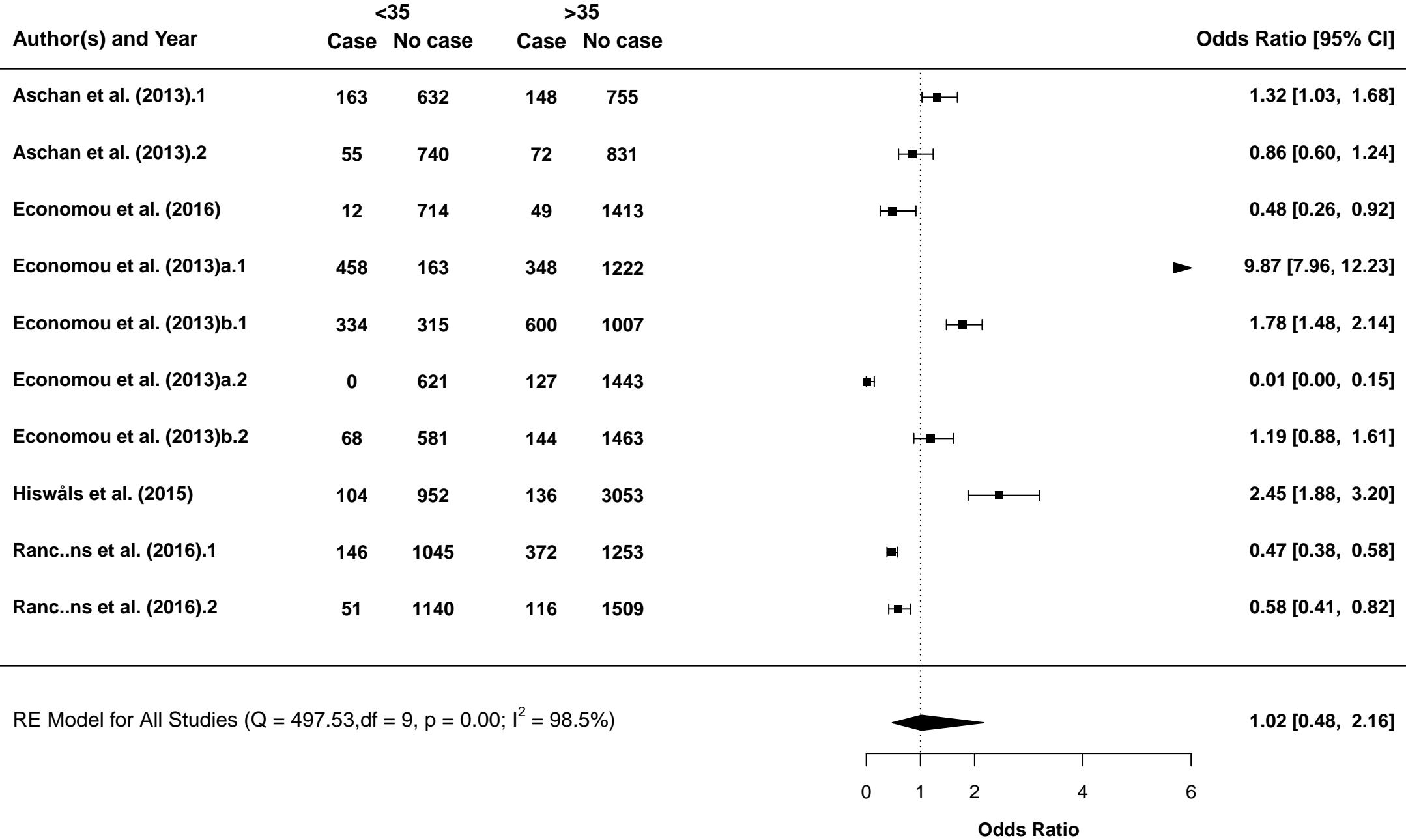

Supplement: Supplementary file 1 [file ijerph-17-04115-s001.zip › Supplementary data/Figures/Figure S3. Age up to 35 years for all suicidality forest plot.pdf]

Edad 35–65

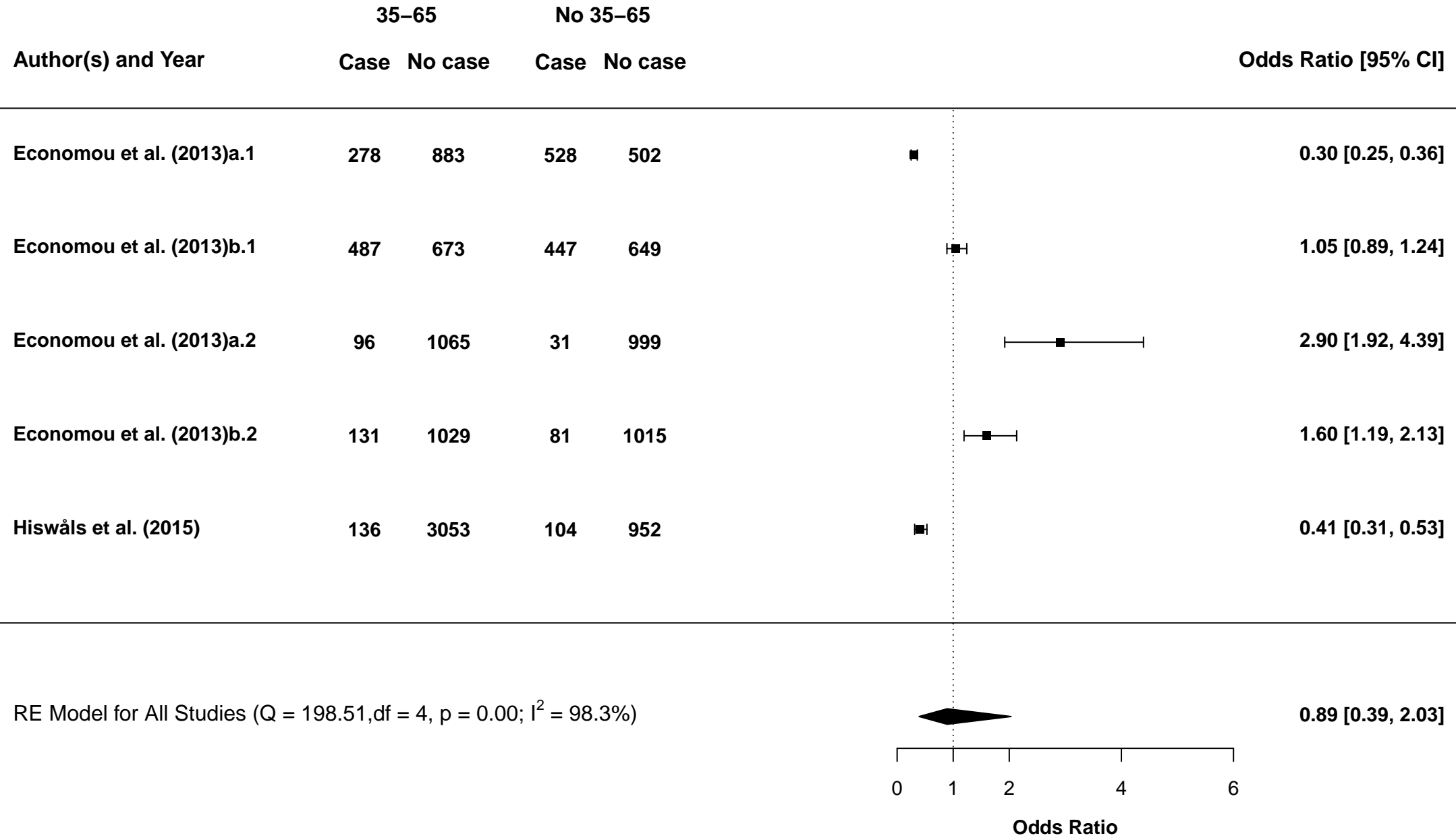

Supplement: Supplementary file 1 [file ijerph-17-04115-s001.zip › Supplementary data/Figures/Figure S4. Age between 35 and 65 years for all suicidality forest plot.pdf]

# Edad > 65

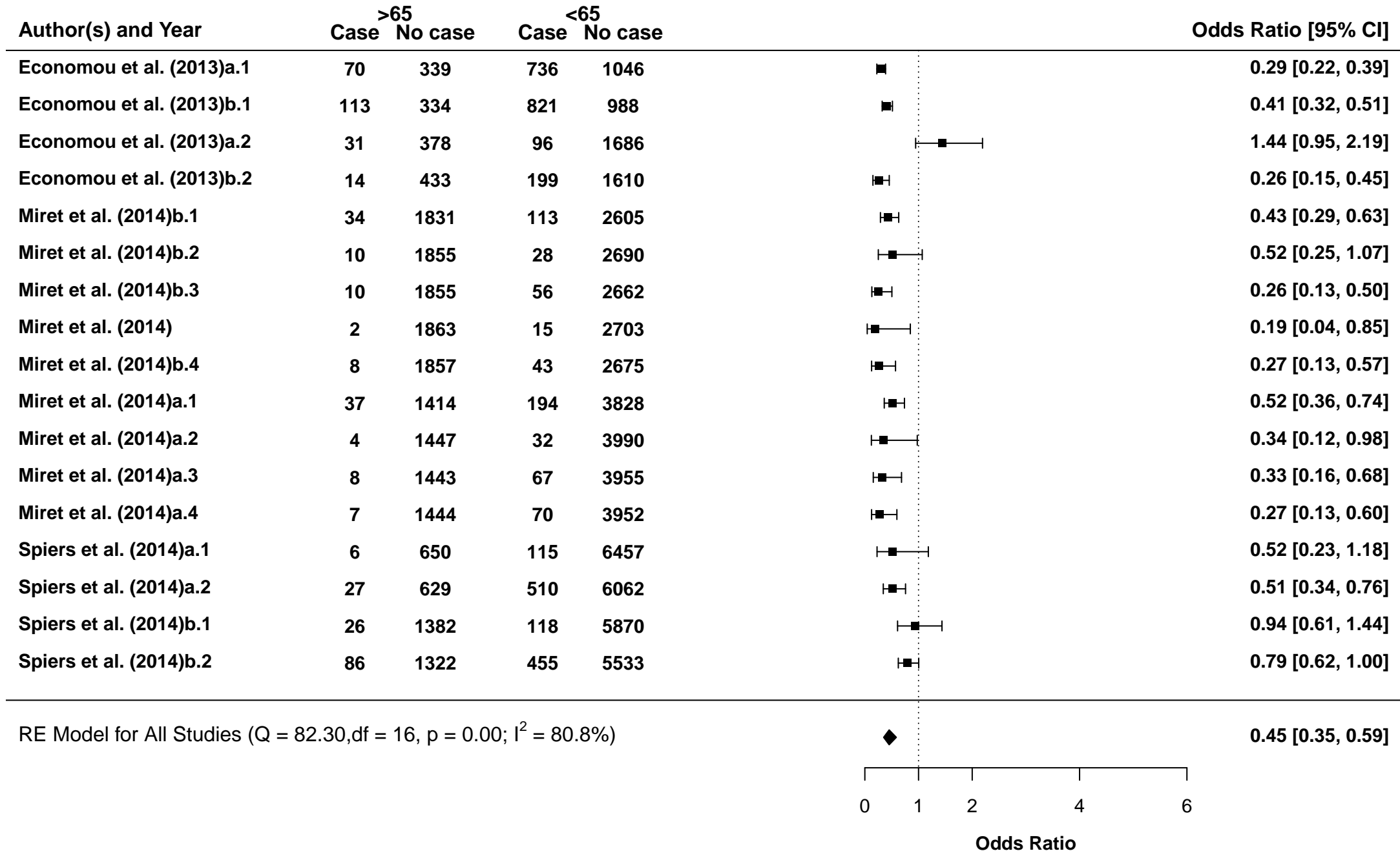

Supplement: Supplementary file 1 [file ijerph-17-04115-s001.zip › Supplementary data/Figures/Figure S5. Age over 65 years for all suicidality forest plot.pdf]

## Estado civil

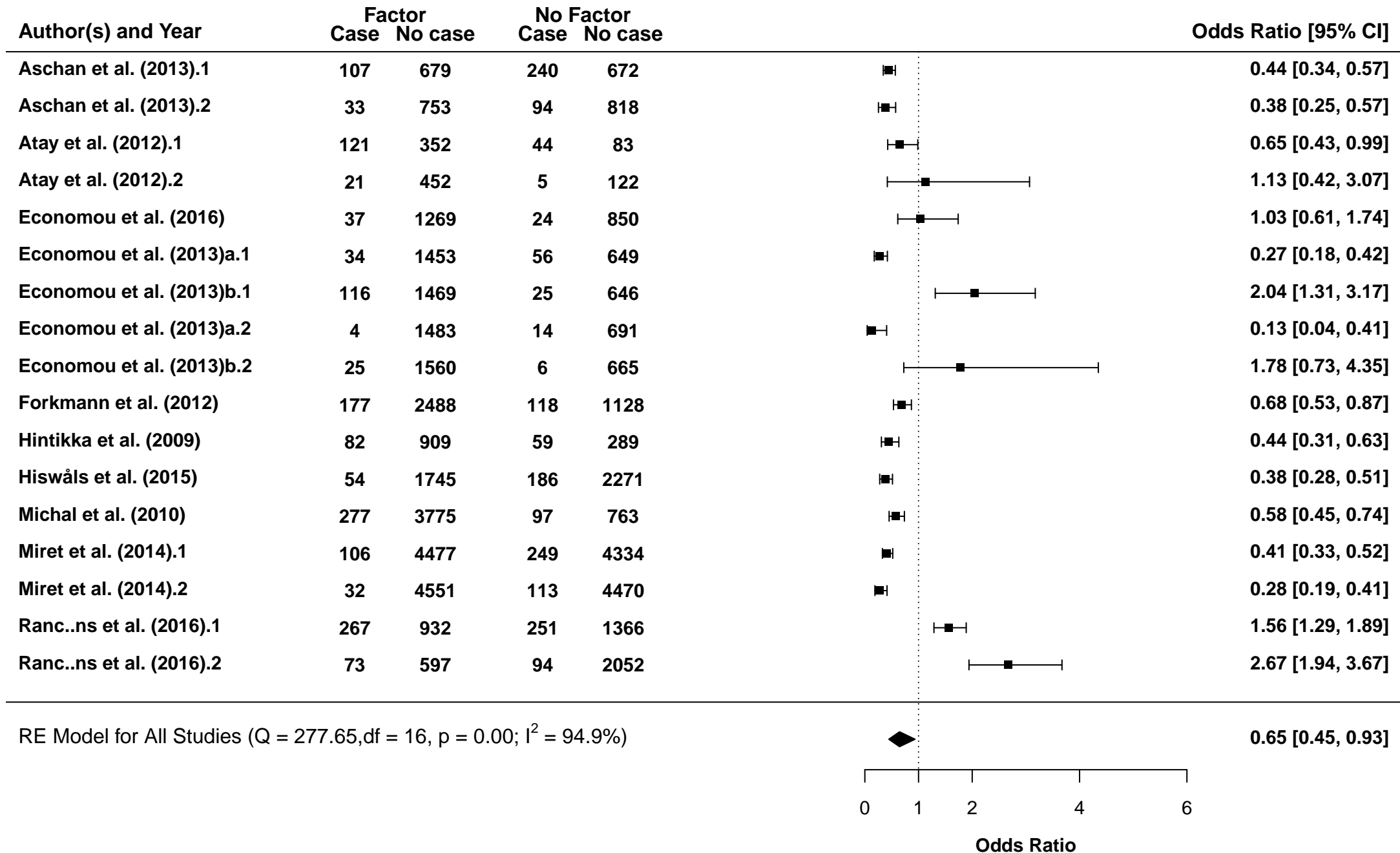

Supplement: Supplementary file 1 [file ijerph-17-04115-s001.zip › Supplementary data/Figures/Figure S6. Relationship status for all suicidality forest plot.pdf]

# Urbano– rural

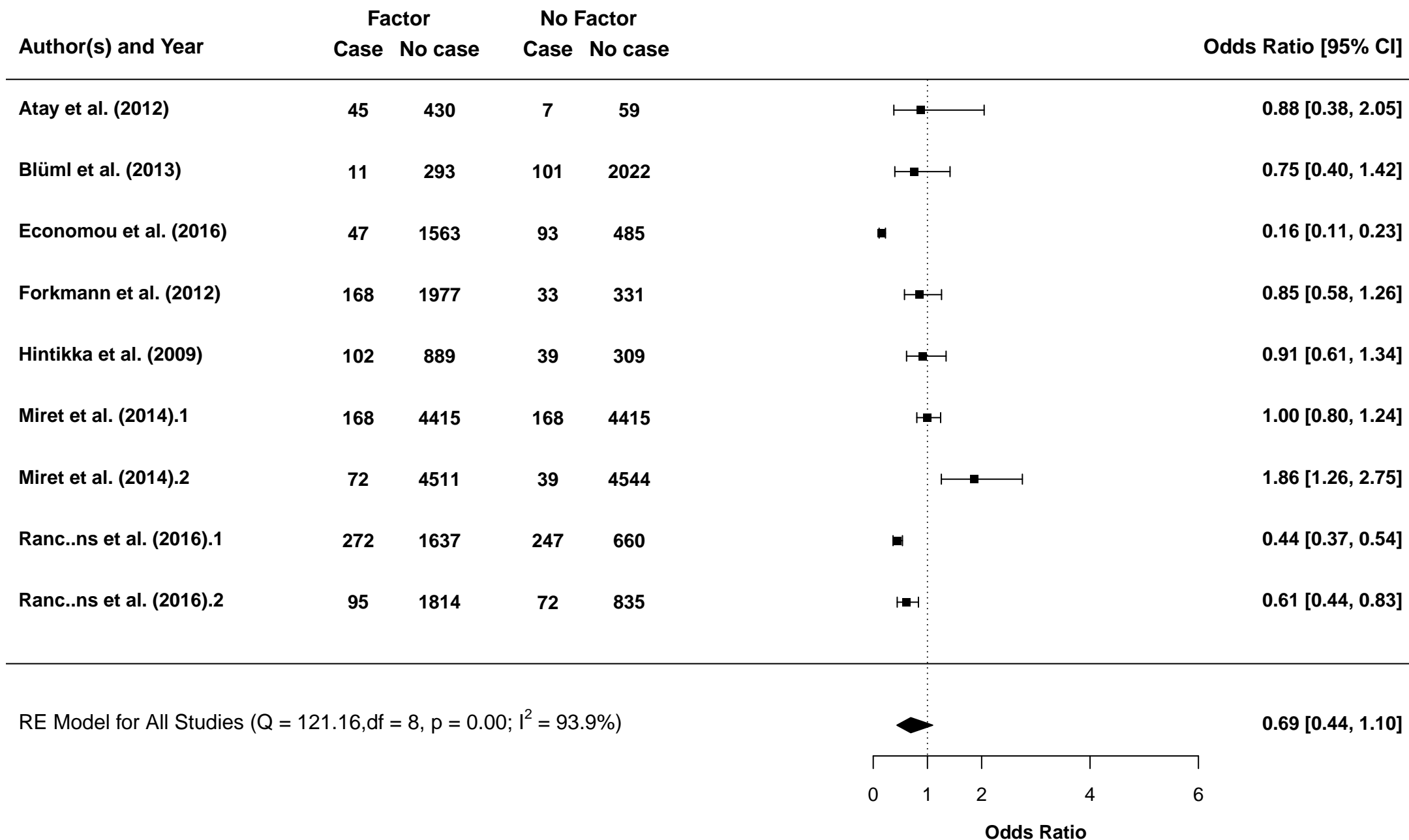

Supplement: Supplementary file 1 [file ijerph-17-04115-s001.zip › Supplementary data/Figures/Figure S7. Residential setting for all suicidality forest plot.pdf]

migrantes

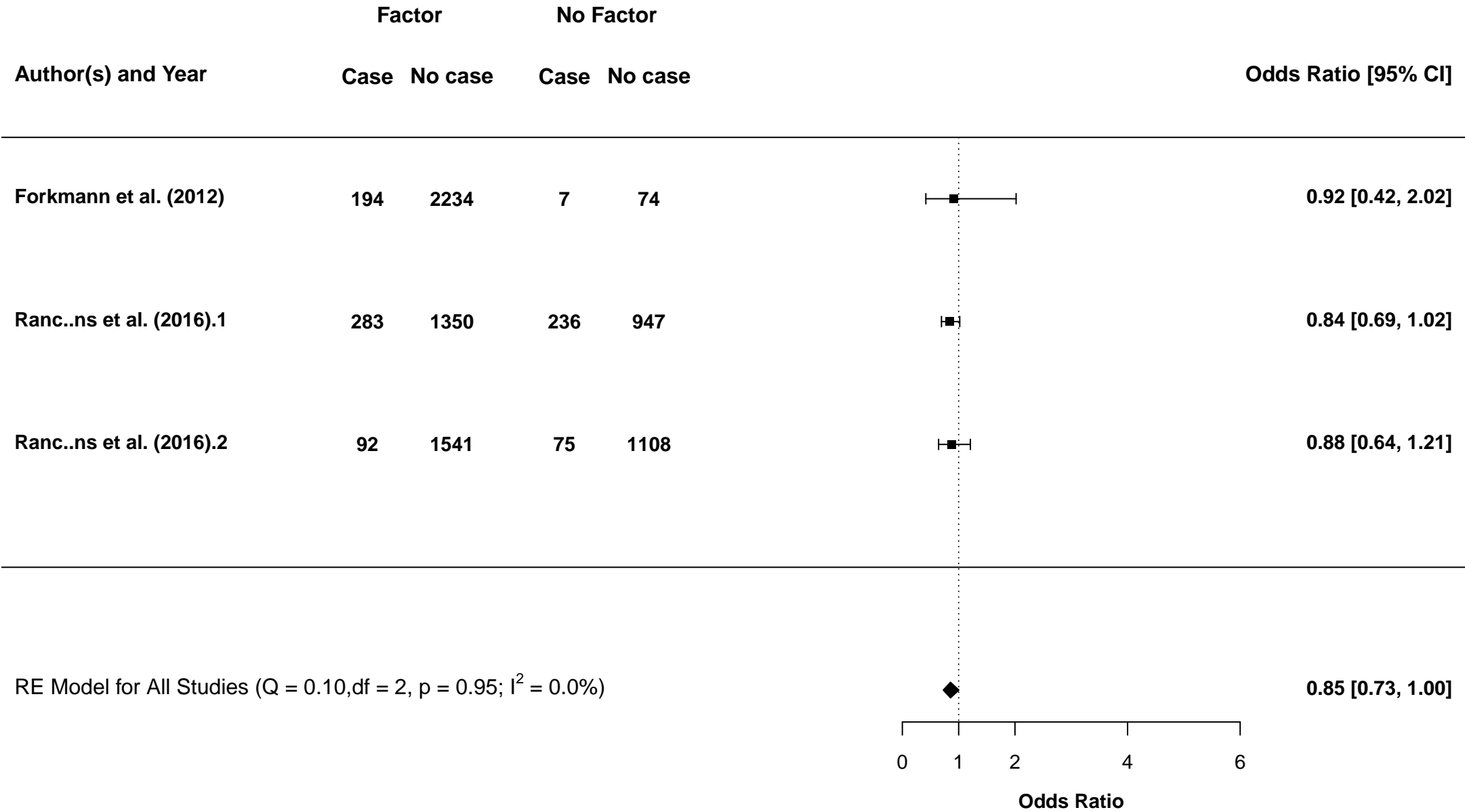

Supplement: Supplementary file 1 [file ijerph-17-04115-s001.zip › Supplementary data/Figures/Figure S8. Nationality for all suicidality forest plot.pdf]

estudios

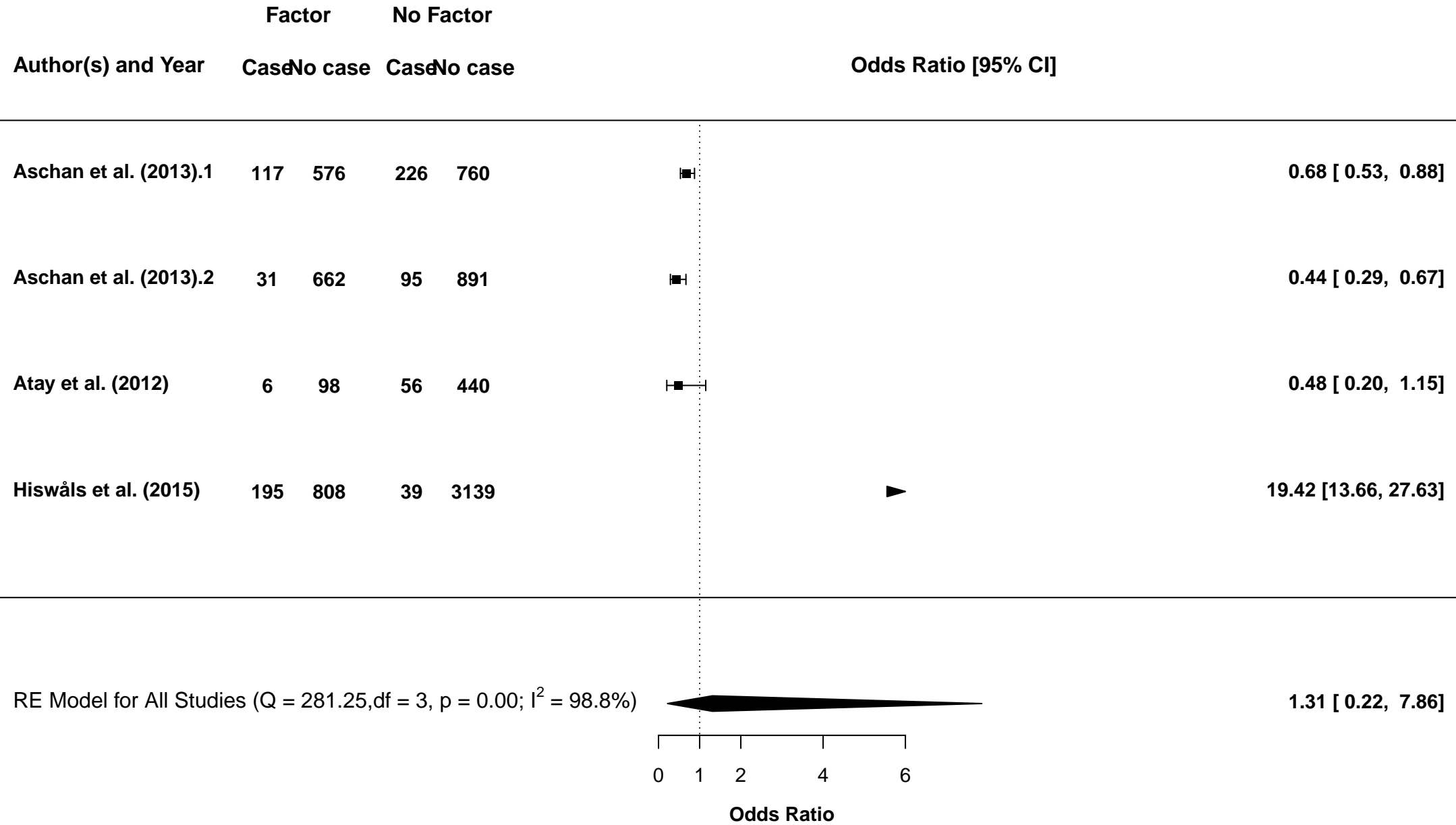

Supplement: Supplementary file 1 [file ijerph-17-04115-s001.zip › Supplementary data/Figures/Figure S9. Education for all suicidality forest plot.pdf]
